# Cross-bridge mechanics estimated from skeletal muscles’ work-loop responses to impacts in legged locomotion

**DOI:** 10.1038/s41598-021-02819-6

**Published:** 2021-12-08

**Authors:** Kasper B. Christensen, Michael Günther, Syn Schmitt, Tobias Siebert

**Affiliations:** 1 Motion and Exercise Science, University of Stuttgart, Allmandring 28, 70569 Stuttgart, Germany; 2 Computational Biophysics and Biorobotics, Institute for Modelling and Simulation of Biomechanical Systems, University of Stuttgart, Nobelstraße 15, 70569 Stuttgart, Germany; 3 Stuttgart Center for Simulation Science (SC SimTech), University of Stuttgart, Pfaffenwaldring 5a, 70569 Stuttgart, Germany

**Keywords:** Biomechanics, Nonlinear dynamics, Bioenergetics, Animal behaviour

## Abstract

Legged locomotion has evolved as the most common form of terrestrial locomotion. When the leg makes contact with a solid surface, muscles absorb some of the shock-wave accelerations (impacts) that propagate through the body. We built a custom-made frame to which we fixated a rat (*Rattus norvegicus*, Wistar) muscle (*m. gastrocnemius medialis and lateralis*: GAS) for emulating an impact. We found that the fibre material of the muscle dissipates between 3.5 and $23\,\upmu \hbox {J}$ ranging from fresh, fully active to passive muscle material, respectively. Accordingly, the corresponding dissipated energy in a half-sarcomere ranges between 10.4 and $68\,z\hbox {J}$, respectively. At maximum activity, a single cross-bridge would, thus, dissipate 0.6% of the mechanical work available per ATP split per impact, and up to 16% energy in common, submaximal, activities. We also found the cross-bridge stiffness as low as $2.2\,\hbox {pN}\,\hbox {nm}^{-1}$, which can be explained by the Coulomb-actuating cross-bridge part dominating the sarcomere stiffness. Results of the study provide a deeper understanding of contractile dynamics during early ground contact in bouncy gait.

## Introduction

For any type of terrestrial locomotion, the common working condition is the active contraction of skeletal muscles, which, in turn, generates skeletal movement through space. In legged locomotion, repulsion from a surrounding solid is required, inducing shock-wave-like accelerations (i.e., impact responses) to the system via the bones to the muscles and joints at touch-down (TD)^1,2^. A course of action that, in turn, causes the muscle material to wobble^1–4^.

According to the muscle-tuning paradigm^5^, changes in muscle activity alter the mechanical properties of the muscle during the impact^6^, therefore, affect both frequency and damping coefficient of its vibrations after TD^1^. Following the theory, the muscle can adjust the damping of its eigenfrequency vibrations after TD^5,7^. Damping of oscillations superposed to muscle contraction results in a dissipation of mechanical energy. In whole muscles, Ettema et al.^8^ calculated the energy dissipated in small-amplitude sinusoidal work-loops (ranging from 5 to 180 Hz) of rat gastrocnemius medialis. In the 5–180 Hz frequency range, the corresponding energy dissipated decreased from 55 to $40\,\upmu \hbox {J}$ in fully activated muscle^8^. However, using this experimental approach, oscillations are imposed on the distal tendon that differ from in-vivo muscle wobbling responses induced by impacts. While muscle material is commonly associated with visco-elastic properties^1,8,9^, most muscle models disregard muscle inertia^10,11^ and consequently ignore the fundamental wobbling mass behaviour of skeletal muscles in impact situations. To our knowledge, there are no experimental data of directly measured damping strengths and energy dissipation associated with muscle wobbling in response to an impact.

By experimentally emulating rat leg impacts during running at $1\,\hbox {m s}^{-1}$^12,13^, we showed^14^ that the muscles must be both maximally activated and non-fatigued to prevent forcible cross-bridge detachment during impacts. We also found that with decreased muscle activity, the high impacts induced by these experiments caused high muscle fibre strains during wobbling^14^. Higher fibre strains were associated with increasing contributions of passive, elastic elements^14^, which complicated the examination of cross-bridge and non-cross-bridge contributions to fibre stiffness at submaximal activities. One possibility to reduce fibre stain is to lower impact intensities by lowering the falling height in the wobbling experiments. Lower fibre strains can then be associated with negligible passive stiffnesses. With this, it is possible to decouple the identification of properties of the passive, connective tissue from those of the cross-bridges. However, to explain microscopic sarcomere properties (e.g. cross-bridge stiffness) based on macroscopic wobbling measurements during impact requires the application of muscle models.

Based on muscle fibre experiments, Fusi et al.^15^ determined cross-bridge stiffnesses and strains using a muscle model consisting of myofilament stiffness *in-series* with the stiffness of the cross-bridge ensemble. In their model, the force generated by a single cross-bridge is assumed a constant, with an attributed constant deflection. Thus, the overall cross-bridge stiffness scales linearly with the number of attached myosin heads. An alternative for determining cross-bridge stiffnesses is the model from Günther et al.^16^. This model can reproduce the early half-sarcomere force recovery phase following rapid step-in-length experiments (T2 curve). According to their model, the ensemble of cross-bridges is *in-series* with a collective of passive stiffnesses, denoted there as a combined myosin head and myofilament stiffness. The cross-bridge itself is divided into a catalytic domain and a light chain domain that can rotate, actuated by a Coulomb force drive, with respect to the catalytic domain. In contrast to Fusi et al.^15^, the force–length relation of this cross-bridge drive is non-linear as it depends on the properties of the repulsing Coulomb force generated within the catalytic domain.

Here, we continue our work^14^ to gain further insight into muscle wobbling during the first few milliseconds after TD in legged locomotion. By reducing the impact, compared to our former study, we aim to better understand damping and energy dissipation of the whole muscle and the fibre material during wobbling. Therefore, we calculated stiffnesses, damping coefficients, and the energy dissipated during work-loops in the range from passive to fully activated muscle, and then scaled these parameters to the half-sarcomere level. Secondly, we aim to probe the predictions of cross-bridge stiffness values by half-sarcomere models: the first by Fusi et al.^15^, and the second by Günther et al.^16^. We probe these models’ potentials to explain, by essential cross-bridge parameters, a muscle’s overall response to an impact.

## Results

### Results from whole muscle experiments

**Figure 1:**
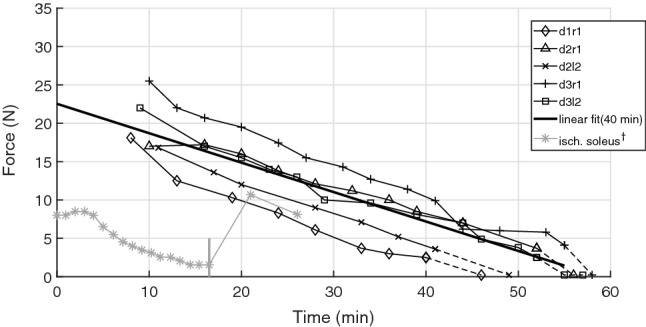
Decline of isometric muscle force (${\varvec{F}}$) at TD versus time after muscle extraction. Only trials with TD force of at least 95% of its isometric value (converged force before or after TD) were included. The solid line is a linear fit to all data points below 40 min extrapolated back to $t=0$. $^{\dag }$The measured isometric force in cat *m. soleus* in response to induced ischaemia from Mortimer et al.^17^. In the shown trial, they stimulated the cat muscle with single twitches ($*$) under ischaemic conditions, whereafter blood flow was returned (grey, vertical line at 16.5 min mark) to recover muscle force ($*\,>$  20  min). In a similar study, the isometric force in rabbit *m. anterior tibialis* recovered 87 % of the measured maximal isometric force after 1 h of ischaemia^18^.

By linear extrapolation of the fatigue trend in our present data back to $t=0$, we estimated an average in-vivo value of $F_{max}\,=\,23\,\hbox {N}$ of maximum isometric GAS force (Fig. 1). In the passive trials, i.e. the non-stimulated muscles, the median of the passive muscle forces measured by the force transducer was 0.25 N. We found that all present experiments had an almost constant peak impact force change in common, with a mean value of $\Delta F\,=\,\,0.20\,\hbox {N}\,\pm \,0.03$.

**Figure 2:**
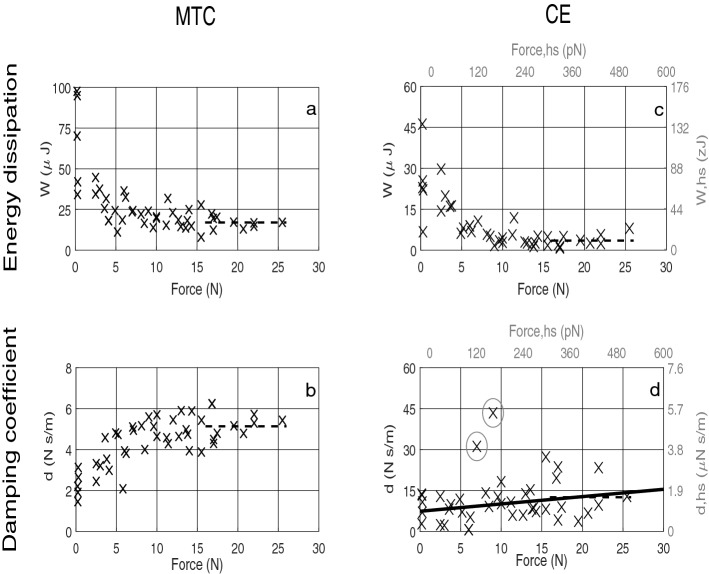
Energy dissipated and viscous damping coefficient of MTC and CE for all isometric and passive force states. (**a**–**d**) Data in each trial are calculated for one work-loop, i.e. one oscillation period that spans between TD and the instant when $a_{COM}$ returns closest to zero for the second time. See Fig. 3 for an example of one work-loop in a specific trial. For (**a**–**d**), the dashed, black line is the mean value of all data points $>\,16\,\hbox {N}$: $17\,\upmu \hbox {J}$, $5.1\,\hbox {N}\,\hbox {s}\,\hbox {m}^{-1}$, $3.5\,\upmu \hbox {J}$ | $10.4\,\hbox {zJ}$ and $12.5\,\hbox {N}\,\hbox {s}\,\hbox {m}^{-1}$ | $1.6\,\upmu \hbox {N}\,\hbox {s}\,\hbox {m}^{-1}$, respectively. (**a**) The energy dissipated by the MTC due to internal material friction. (**b**) The viscous damping coefficient calculated for MTC. (**c**) The energy dissipated by the CE, with the right and upper axes giving the work (Eq. 4) and isometric force (Eq. 3) values per half-sarcomere, respectively. (**d**) The viscous damping coefficient calculated for the CE, with the right and upper axes giving the damping coefficient (Eq. 5) and isometric force (Eq. 3) values per half-sarcomere, respectively. Due to the indistinct trend in (**d**), a linear fit was added. In (**d**), the circles indicate data that were considered outliers and excluded from the fit.

**Figure 3:**
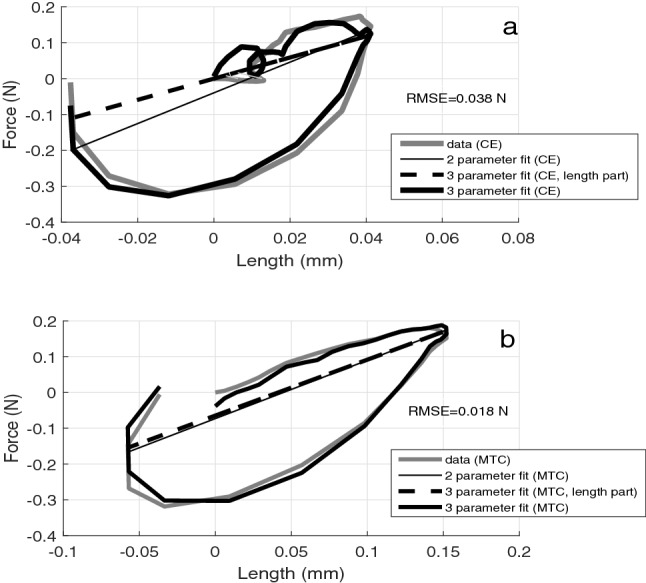
The work-loops of CE (**a**) and MTC (**b**) in one passive exemplary trial. The solid, dark-grey loops are the $L_{CE,0}$ or $L_{MTC,0}$ responses, respectively, to $\Delta F$. The solid, thin, black line is the respective linear 2-parameter (force (length)) fit to the data. The solid, black loops depict the respective 3-parameter fits to the data, using the parameters $k_{i}$, $b_{i}$, $d_{i}$ of each the function $F_{i}(L_{i},\dot{L}_{i})$ (see Eq. 1), which linearly depends on length $L_{i}$ and time rate of length change $\dot{L}_{i}$; the dashed, black line is the respective length-dependent contribution. Both work-loops encompass one oscillation period that spans between TD and the instant when $a_{COM}$ returns closest to zero for the second time (Supplementary Fig. S1). The Root Mean Square Error (RMSE) for the 3-parameter fit to data in (**a**,**b**) is 0.038 N and 0.018 N, respectively.

In Fig. 2, we estimated the energy dissipated, as the area enclosed per one work-loop (Examples in Fig. 3), and the respective damping coefficient (inferred from Eq. 1) of the muscle-tendon complex (MTC) and the contractile element (CE). Here we assume that the MTC consists of two in-series units: muscle fibre material and tendon material (including aponeurosis), where the fibre material part of the MTC is labelled CE. At $F_{max}$, the MTC and CE dissipated on average $17\,\upmu \hbox {J}$ (Fig. 2a) and $3.5\,\upmu \hbox {J}$ (Fig. 2c), respectively. The latter values increased to $70\,\upmu \hbox {J}$ (Fig. 2a) and $23\,\upmu \hbox {J}$ (Fig. 2c), respectively, in the passive experiments (both passive median values). Across all trials, the energy dissipated by the CE and MTC decreased with isometric force, which was in contrast to the found damping coefficients that increased along with the isometric force. In more detail, MTC damping coefficients increased from around $2.2\,\hbox {N}\,\hbox {s}\,\hbox {m}^{-1}$ in a passive muscle to about $5.1\,\hbox {N}\,\hbox {s}\,\hbox {m}^{-1}$ in active muscle above 10 N (Fig. 2b). With regard to the CE damping coefficient, the latter trend was more unclear due to data scatter, though, the damping coefficient seemed to increase from $\approx \,9\,\hbox {N}\,\hbox {s}\,\hbox {m}^{-1}$ in a passive muscle to $\approx \,12.5\,\hbox {N}\,\hbox {s}\,\hbox {m}^{-1}$ in active muscle at $F_{max}$ (Fig. 2d).

### Parameter scaling to the half-sarcomere level

Dissipated CE energy and the CE damping coefficient were scaled to a half-sarcomere, using Eqs. (4) and  (5), respectively (right and upper axes in Fig. 2c,d). Accordingly, the energy dissipated in the CE ranged from 68 $z\hbox {J}$ in the median passive half-sarcomere to 10.4 $z\hbox {J}$ at $F_{max}$, and the CE damping coefficient was $\approx \,1.1\,\upmu \hbox {N}\,\hbox {s}\,\hbox {m}^{-1}$ in a passive half-sarcomere and $\approx \,1.6\,\upmu \hbox {N}\,\hbox {s}\,\hbox {m}^{-1}$ at $F_{max}$.

**Figure 4:**
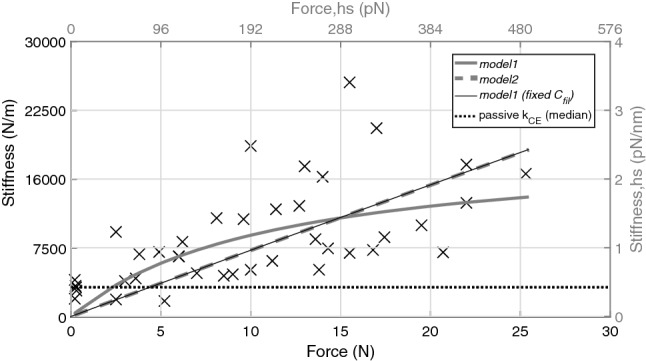
Contractile element stiffness (${{k}}_{{{{CE}}}}$). Trial specific $k_{CE}$ values were inferred from a 3-parameter fit (Eq. 1), with information from one oscillation period after TD (Supplementary Fig. S1). $k_{CE}$ is also given as scaled to the dimension of one representative half-sarcomere ($k_{hs}$, right axis, see Eq. 2). The correspondingly scaled isometric force per half-sarcomere (Eq. 3) is given on the upper axis. The solid and dashed grey lines represent the *model1* and *model2* fits (see Table 2), respectively. The dotted, horizontal, black line indicates the median of passive $k_{CE}$ values. The thin, black line underlying the *model2* fit, is the *model1* fit with only the parameter $\Delta L_{CE}$ open (fixed $C_{fil}\,=\,0.0067\,\hbox {nm}\,\hbox {pN}^{-1}$).

Figure 4 shows that the median CE stiffness (median $k_{CE}$) in passive muscle ($3200\,\hbox {N}\,\hbox {m}^{-1}$; dotted, horizontal, black line), was lower than in almost all active trials (cross (x) scatter > 1 N). In the active trials, $k_{CE}$ ranged from $\approx \,4200\,\hbox {N}\,\hbox {m}^{-1}$ at $F\,=\,1\,\hbox {N}$ to $\approx \,13{,}800\,\hbox {N}\,\hbox {m}^{-1}$ at $F\,=\,F_{max}\,=\,23\,\hbox {N}$ (for linear fits of MTC and CE data $>\,1\,\hbox {N}$, see Supplementary Fig. S4).

With the use of GAS dimensions from Table 1, we scaled our stiffness and force (Eq. 1) values to half-sarcomere level: stiffness ($k_{hs}$, Eq. 2) and isometric force ($F_{hs}$, Eq. 3), respectively. For $F_{max}\,=\,23\,\hbox {N}$ (Fig. 1), we would predict the isometric maximum $F_{hs}$ to be 445 pN, which is practically the same value as estimated by others^19^ under the same assumptions as made here (Supplementary Text S4).

**Table 1:** Anatomical data given as the mean value ± standard deviation.

Description	Symbol	Data	Unit	Source
Animal mass		406 ± 6	g	Measured
GAS mass		1.9 ± 0.2	g	Measured
GAS length at $90^{\circ }$	$L_{GAS,90^{\circ }}$	41 ± 1	mm	Measured
GAS length in frame	$L_{GAS,0}$	43	mm	$L_{GAS,90^{\circ }}+2^{\dag }$
Belly length		31	mm	$L_{GAS,0}$ $-$ $L_{tendon,0}$
Reference length	$L_{CE,0}$	7.5 ± 1.7	mm	Measured
Proximal tendon length	$L_{prox,0}$	2$^{\dag }$	mm	Literature
Distal tendon length	$L_{dist,0}$	10.1 ± 0.5	mm	Measured
Total tendon length	$L_{tendon,0}$	12	mm	$L_{prox,0}$+$L_{dist,0}$
Maximum belly ACSA	$A_{CE,max,0}$	96 ± 5	mm$^{2}$	Measured
Minimum belly ACSA	$A_{CE,min,0}$ $^\mathbf{\ddag }$	81 ± 16$^\mathbf{* }$	mm$^{2}$	Measured
Average belly ACSA	$A_{CE,avr,0}$	86	mm$^{2}$	$\frac{A_{CE,0,max}+A_{CE,0,min}}{2}$
Tendon ACSA	$A_{tendon,0}$	1.9 ± 0.7	mm$^{2}$	Measured

We calculated the anatomical cross-sectional area (ACSA) right before TD by assuming that the belly had the geometrical shape of a half-ellipse.

$^{\dag }$The 2 mm added to measured $L_{GAS,90^{\circ }}\approx L_{opt}$ were inferred from literature^47,48^.

$^\mathbf{\ddag }$ $A_{CE,min,0}$ was measured $\approx$ 8 mm distal, along the muscle belly, to where $A_{CE,0,max}$ was measured, both in passive muscle state.

$^\mathbf{* }$ The relatively large SD in $A_{CE,0,min}$ is due to one outlier geometry.

### Results of model calculations

Using the half-sarcomere values of $F_{hs}$ and $k_{hs}$ we fitted two different model ideas (see “8Model ideas (short version)”) to the data to better understand the underlying half-sarcomere mechanics in response to an impact. The solid, grey line in Fig. 4 is a fit of *model1* by Fusi et al.^15^ to our scaled $k_{hs}$ data with passive trials excluded, which predicts a stiffness of $1.8\,\hbox {pN}\,\hbox {nm}^{-1}$ at 23 N and approaches zero on a slightly curved course, as the isometric force approaches zero. The latter is slightly different from the fitted course of *model2* by Günther et al.^16^ (the dashed, grey line in Fig. 4), which appears practically linear, with a slightly higher stiffness at $23\,\hbox {N}$ ($2.2\,\hbox {pN}\,\hbox {nm}^{-1}$) than predicted by *model1*. The fitted parameter values for both models are given in Table 2.

**Table 2:** Parameter estimations.

Model	${{c_{3}}}$ (nm)	${{\Delta }} {{L}}_{{{CB}}}$ (nm)	${{C_{fil}}}$ ($\hbox {nm}\,\hbox {pN}^{-1}$)
*Model1*	–	85.7	0.4
*Model1*$^\mathbf{* }$	–	198	0.0067$^{{*}}$
*Model2*	1.2	–	–

In *model2*, the parameter $c_{3}$ represents the pole (at $L_{CB}\,=\,-c_{3}$) in the non-linear cross-bridge force–length relation $F_{CB}(L_{CB})$ (Coulomb drive *in series* to the serial elastic part representing S1, S2 and filaments), which is used to estimate $k_{CB}$, and eventually $k_{hs}$. In *model1*, the parameter $\Delta L_{CB}$ (nm) represents the average elongation at a fixed force of each cross-bridge acting *in series* to the filament part with compliance $C_{fil}$. The parameter values of both *model1* and *model2* were determined with the Matlab curve fitting tool ‘cftool’. If the maximum isometric force of a half-sarcomere $F_{CB,max}$ is 445 pN, as estimated in this paper, then the original parameter values for *model1* ([$\Delta L_{CB}\,=\,1.56\,\hbox {nm}$ and $C_{fil}\,=\,1.77\,\hbox {nm}\,T_0^{-1}$]^15^) would translate to $k_{CB}\,=\,285\,\hbox {pN}\,\hbox {nm}^{-1}$ ($\frac{445\,pN}{1.56\,nm}$) and $k_{fil}\,=\,251\,\hbox {pN}\,\hbox {nm}^{-1}$ ($\frac{445\,pN}{1.77\,nm}$) at $F_{CB,max}$.

$^\mathbf{* }$ $\Delta L_{CB}$ (nm) in *model1* estimated with a fixed $k_{fil}\,=\,150\,\hbox {pN}\,\hbox {nm}^{-1}$ value ($C_{fil}\,=\,\frac{1}{k_{fil}}=\frac{1}{150\,pN\,nm^{-1}}\,=\,0.0067\,nm\,pN^{-1}$).

## Discussion

Our determined damping coefficient *d* can be interpreted to represent, together with stiffness *k* and mass *m*, a GAS MTC that responds visco-elastically to the impact by a damped harmonic oscillation around an operating point at the isometric force level *F*. We assess the damping strength by comparing *d* with the critical damping coefficient $d_{crit}\,=\,2\sqrt{k m}$, i.e., by calculating $\zeta \,=\,\frac{d}{d_{crit}}$. As can be seen in Fig. 2b, the inferred damping coefficients at $3\,\hbox {N}, 5\,\hbox {N}$ and $7\,\hbox {N}$ are $3\,\hbox {N}\,\hbox {s}\,\hbox {m}^{-1}$, $4\,\hbox {N}\,\hbox {s}\,\hbox {m}^{-1}$ and $4.5\,\hbox {N}\,\hbox {s}\,\hbox {m}^{-1}$, respectively. The corresponding stiffnesses are $1930\,\hbox {N}\,\hbox {m}^{-1}$, $2070\,\hbox {N}\,\hbox {m}^{-1}$ and $2240\,\hbox {N}\,\hbox {m}^{-1}$ (Supplementary Fig. S4), and the GAS mass is on average 1.9 g (Table 1). From this, we find the damping ratio $\zeta \,=\,1$ for the MTC at $F\,=\,5\,\hbox {N}$, as well as $\zeta \,=\,0.8$ and $\zeta \,=\,1.1$ for $2.5\,\hbox {N}$ and $7.5\,\hbox {N}$, respectively. Thus, the MTC is critically damped at 20% of $F_{max}$ (Fig. 1), under-damped for activity lower 20%, and slightly over-damped for higher activity levels.

If the muscle force directly relates to muscle activity^19,20^ (see also stiffness fits in Supplementary Fig. S4), then the 20% of $F_{max}$ in Fig. 1 is the same as the pre-activation in human GAS before TD (20%)^21^. Accordingly, the number of cross-bridges before an impact relates to soft tissue vibration control in the first few milliseconds after TD. Several studies have experimentally investigated the association between muscle activation and almost critical damping of muscle vibration in response to an impact^1,5,6^, which lead to the muscle-tuning paradigm^5^. However, a limitation of conducting impact experiments with human subjects is the inability to decouple any effect of leg geometry, joint compliance and muscle activity. Conversely, a benefit of our ex-vivo setup is the direct control over GAS isometric force generation and the impact situation: soft tissue MTC properties and conditions affecting its vibration responses can be manipulated independently of the impact strength (falling height).

**Figure 5:**
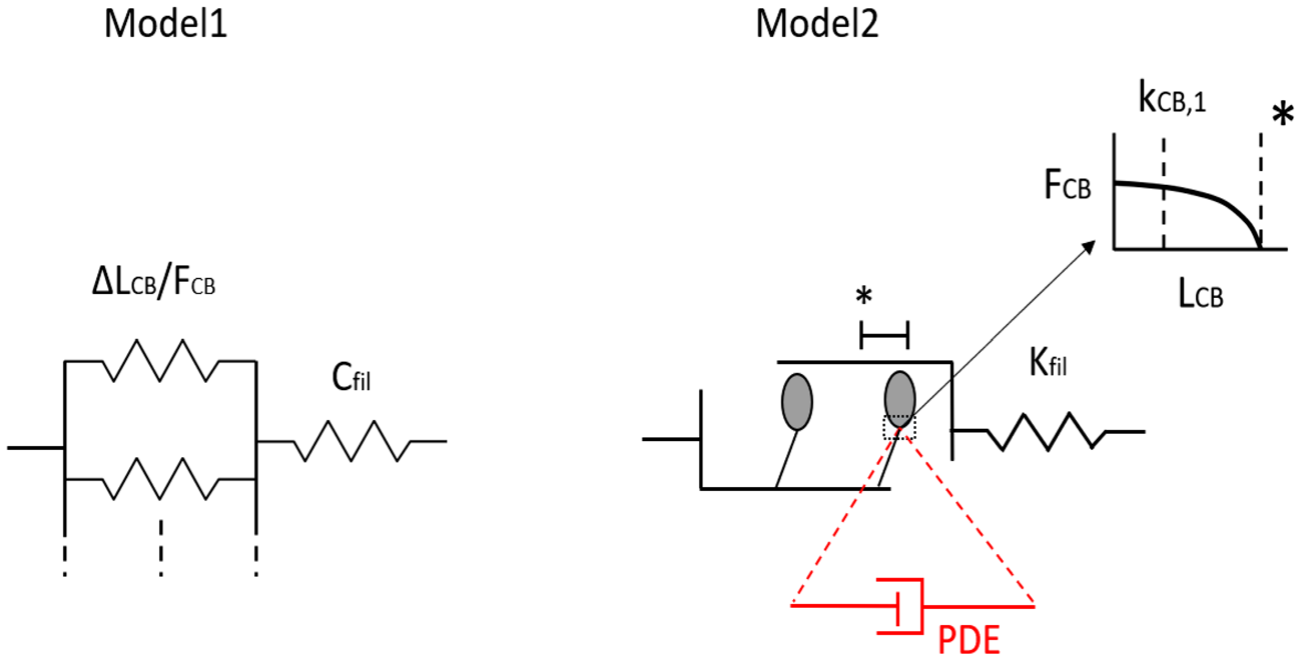
Elements that, according to *model1* and *model2*, contribute to the half-sarcomere stiffness $k_{hs}$. In the elastic *model1*, the myofilament compliance ($C_{fil}$) is in series with stiffnes $k_{CB}$ the number of attached *in-parallel* myosin heads (cross-bridges, CB). The force generated by a single cross-bridge is assumed to be a constant, with an associated constant deflection ($\Delta L_{CB}$). Thus, the stiffness of the ensemble of cross-bridges only ($k_{CB}\,=\,\frac{F_{CB}}{\Delta L_{CB}}$) scales linearly with the number of attached myosin heads. In the non-linear, visco-elastic *model2*, the half-sarcomere stiffness $k_{hs}$ is likewise determined by the number of *in-parallel* attached myosin heads, with each head’s driving non-linear force–length relation $F_{CB}(L_{CB})$ depicted in the top right inset, and a collective of *in-series* passive stiffnesses denoted myofilament stiffness ($k_{fil} = \frac{1}{C_{fil}}$), see Eq. (8). We determined $k_{CB} (F_{CB})$ (Eq. 7) under the assumption that $L_{CB}\,=\,L_{CB,opt}$ = 7 nm, i.e., $F_{CB}=F_{CB,max}$. See Supplementary Fig. S5 for $F_{CB}(L_{CB}$) as determined with original model parameters. Note that, to compare *model1* and *model2*, we excluded the visco-elastic PDE from *model2* (accordingly, PDE is marked in red). $^\mathbf{* }$ The dashed line at the asterisk marks the end of the work-stroke.

In contrast, the CE part is always slightly over-damped across the whole isometric force range, as we find $\zeta \,=\,1.3$ ($d\,=\,7.6\,\hbox {N}\,\hbox {s}\,\hbox {m}^{-1}$, $k\,=\,4250\,\hbox {N}\,\hbox {m}^{-1}$) at $F\,=\,1\,\hbox {N}$ and $\zeta \,=\,1.2$ ($d\,=\,12.5\,\hbox {N}\,\hbox {s}\,\hbox {m}^{-1}$, $k\,=\,13{,}800\,\hbox {N}\,\hbox {m}^{-1}$) at $F\,=\,F_{max}\,=\,23\,\hbox {N}$. This suggests, that the CE system is to return both as smoothly and as quickly at the same time to its equilibrium state, or it may be important for the CE not to overshoot its equilibrium state. The latter may potentially have higher importance as the force–length relation of the work-stroke is non-linear, with even decreasing stiffness of a cross-bridge if the sarcomere is elongated (see inset at the right top in Fig. 5).

In response to an almost constant force change, $\Delta F\,=\,0.2\,\hbox {N}$, the energy dissipated by the MTC at $F_{max}$ was calculated as $17.0\,\upmu \hbox {J}$ (Fig. 2a). Therefore, the *m. gastrocnemius medialis* head would (scaled by ACSA) roughly account for $8.5\,\upmu \hbox {J}$, which is only 21% of the $40\,\upmu \hbox {J}$ previously estimated for Wistar *m. gastrocnemius medialis* in one work-loop at $50\,\hbox {Hz}$ with $1\,\hbox {N}$ peak-to-peak force for one oscillation period^8^. This $1\,\hbox {N}$ peak-to-peak force would correspond to a $0.5\,\hbox {N}$ force change because our force change found covers only half of a full oscillation period. In contrast, we found $\Delta F\,=\,0.2\,\hbox {N}$ for the whole gastrocnemius, of which *m. medialis* would then roughly account for $0.1\,\hbox {N}$. This latter value is about 20% of the comparable $0.5\,\hbox {N}$ force change (half oscillation period) in Ettema and Huijing^8^, which is in perfect accordance with the 21% between 8.5 and $40\,\upmu \hbox {J}$.

At $F_{max}$, the energy dissipated by the CE was $3.5\,\upmu \hbox {J}$ (Fig. 2c). Using Eq. (4), and assuming that the maximum number of myosin heads in a half-sarcomere bound at $F_{max}$ is $n_{CB,max}\,=\,90$^19^, the energy dissipated per cross-bridge is $\frac{10.4\,z\text {J}}{90}\,=\,0.12\,z\hbox {J}$ ($1.2\cdot 10^{-22}\,\hbox {J}$) for a fresh and fully stimulated muscle (Fig. 2c). To put 0.12 $z\hbox {J}$ into perspective, the free energy $\Delta G_{ATP}$ available from ATP hydrolysis within a cell is $54\,\hbox {kJ}\,\hbox {mol}^{-1}$ for rabbit psoas (fast-twitch) and $66\,\hbox {kJ}\,\hbox {mol}^{-1}$ for rabbit soleus (slow twitch)^22^, which corresponds to $90\,z\hbox {J}$ and $110\,z\hbox {J}$ per ATP molecule, respectively^23^. Reported values for cross-bridge thermodynamic efficiency, i.e. the fraction of $\Delta G_{ATP}$ converted into work, is around 21% for mouse *m. extensor digitorum longus* (fast) and 45% for tortoise *m. rectus femoris* (slow)^23^. The $\Delta G_{ATP}$ value for mouse *m. extensor digitorum longus* suggests that for a muscle dominated by fast-twitch fibres like GAS, the mechanical work available per one ATP molecule split is around $0.21\,\cdot \,90\,z\hbox {J}\,=\,19\,z\hbox {J}$. Therefore, one impact for the GAS would lead to an 0.6% ($\frac{0.12}{19}$) energy loss per cross-bridge at $F_{max}$, because the myosin is believed to be bound to actin for 450 ms under isometric conditions^24,25^, and the wobbling impact response does not take more than 25 ms (Supplementary Fig. S1). On the other hand, if GAS was pre-activated by only 20% before an impact as in humans^21^, then the energy loss per cross-bridge would be 7.9% for an impact, because the dissipated energy per half-sarcomere at $F\,=\,5\,\hbox {N}$ is about $27\,z\hbox {J}$ (Fig. 2c), and the number of myosin heads bound in a half-sarcomere may be approximately $n_{CB}\,=\,0.2 \cdot n_{CB,max}\,=\,18$. In the latter more realistic case, one cross-bridge would dissipate about $2 \cdot 7.9$% $\approx$ 16% of the mechanical work available ($19\,z\hbox {J}$) due to the impacts, since the stride cycle for a rat hindlimb is 300 ms^26^, which is 150 ms shorter than the myosin-actin bound state. Therefore, our findings suggest that ignoring wobbling in muscle models, especially those emulating legged locomotion^10,11^, can lead to underestimating the energetic costs associated with walking or running up to 16% in the fibre material. Accordingly, at even lower pre-activation, the energy dissipated increases non-linearly (Fig. 2c). Note that with our setup, we restricted the experimental condition to isolated muscles that were vertically oriented, and we solely analysed the muscle’s response to an impact in the vertical direction. The energy dissipated in GAS in-vivo may differ from our findings because of muscle friction with surrounding tissues (e.g. skin, bones or other muscles), or simply because the actual impact shock-wave mode(s) are not restricted to travel almost solely longitudinal to the fibre direction.

It is likely that several structural—such as titin—contributions to passive visco-elasticity act in parallel to the cross-bridges, which contribute to the variations in energy dissipated in passive trials (see Supplementary Text S7). However, due to the low sample size and our setup, our experiments were not suited to resolve such potential single passive contributions across the isometric force range of active muscles.

For better understanding cross-bridge mechanics, we fitted the parameters (Table 2) of two CE models (see “8Model ideas (short version)”), *model1*^15^ and *model2*^16^, to reproduce our measured CE stiffness $k_{CE}$ (Fig. 4).

Regarding *model1*, the best fit of $k_{hs}$ predicted $1.8\,\hbox {pN}\,\hbox {nm}^{-1}$ at $F_{max}$, which yielded parameter values of cross-bride deflection ($\Delta L_{CB}\,=\,85.7\,\hbox {nm}$) and myofilament stiffness ($k_{fil}\,=\,2.5\,\hbox {pN}\,\hbox {nm}^{-1}$) that are factors of 55 and $\frac{1}{34}$, respectively, from earlier model estimations^15^.

As a consequence of the estimated 85.7 nm work-stroke for *model1*, the stiffness of a single cross-bridge would be $0.05\,\hbox {pN}\,\hbox {nm}^{-1}$ if the force for a single cross-bridge is 4 pN^19,24^. Vice versa, if a force for a single cross-bridge were 100 pN, then the cross-bridge would have a realistic^27–29^ stiffness of $1.2\,\hbox {pN}\,\hbox {nm}^{-1}$. Therefore, *model1* can not explain the low $k_{hs}$ values found in these experiments: at least one of the three widely accepted parameter values, work-stroke length^27,30^, force^31,32^, or stiffness^19,28,29^, would be heavily compromised. In accordance with the original model formulation of *model1*, both the cross-bridge stiffness ($k_{CB}$) and $k_{fil}$ are free parameters. However, if $k_{fil}\,=\,150\,\hbox {pN}\,\hbox {nm}^{-1}$ applies as in *model2*, then the work-stroke would be even higher than the estimated $85.7\,\hbox {nm}$. A fixed $k_{fil}\,=\,150\,\hbox {pN}\,\hbox {nm}^{-1}$ would also make the $k_{hs}$ fit of *model1* appear more linear, like that predicted by *model2* (see Fig. 4), due to a then forced change in myofilament compliance ($C_{fil}\,=\,\frac{1}{k_{fil}}$) and cross-bridge deflection $\Delta L_{CB}$ (Table 2).

Contrary to *model1*, *model2* assumes a non-linear force–length relation of the Coulomb-actuated cross-bridge-driving part in the CE, which depends on its pole value ($c_{3}$) in the cross-bridge force–length relation. A change in $c_{3}$ does neither affect the force nor the work-stroke length measured from the cross-bridge’s optimal state. However, $c_{3}$ does change $\frac{dF}{dL}$ with changing cross-bridge position. With $c_{3}\,=\,1.2\,\hbox {nm}$ (Table 2), $k_{hs}$ would be 2.2$\,\hbox {pN}\,\hbox {nm}^{-1}$ at $F_{max}$ (Fig. 4). Under the same $k_{fil}\,=\,150\,\hbox {pN}\,\hbox {nm}^{-1}$ assumption as above, the overall stiffness value of the cross-bridge part $k_{CB}$ for *model2* is $2.2\,\hbox {pN}\,\hbox {nm}^{-1}$ (Eq. 8) at $F_{max}$ ($n_{CB,max}\,=\,90$), practically making $k_{CB}\approx k_{hs}$. The latter stiffness is a factor of 4 from an estimated $k_{CB} \approx n_{CB,max} \cdot 0.1\,{\text {pN}~\text {nm}}^{-1} \approx 9\,\text {pN}\,\text {nm}^{-1}$, which is the overall stiffness of the cross-bridge part at $F_{max}$ when calculated with the parameters given in the original paper (see Fig. 4^16^). For a *model2* $F_{CB}(L_{CB})$ comparison, see Supplementary Fig. S5. The factor of 4 discrepancy between the original stiffness choice for the active fibre material and our found value of 2.2$\,\hbox {pN}\,\hbox {nm}^{-1}$ may be adopted for reconsidering the parameters of the Coulomb force interaction assumed in *model2*^16^, e.g., considering dipole-dipole interaction^33^ or electric permittivity. The difference between originally 9$\,\hbox {pN}\,\hbox {nm}^{-1}$ and our measured 2.2$\,\hbox {pN}\,\hbox {nm}^{-1}$ may also be due to the dynamics inherent to the shock-waves that propagated through the CE in our experiments, which potentially caused some local sarcomere compression. If compression were to occur, then the sarcomeres here could be dominated by the low 0.01–$0.02\,\hbox {pN}\,\hbox {nm}^{-1}$ bending stiffness of the myosin sub-fragment S2^34,35^. That local sarcomere compression can occur seems plausible, because, in rare trials  we observed macroscopic CE shortening to precede elongation, which occurs *after* TD (Supplementary Text S1). However, due to insufficient spatial resolution, an adequate examination has not been possible so far.

Despite *model2* appears to better explain our finding here, previous applications of *model1* have been proven very robust^15,36,37^, with $k_{hs}$ either inferred from rapid step-in-length experiments^36^ or 4 nm peak-to-peak oscillations per half-sarcomere at 4000 Hz^15,37^. However, according to step-in-length, or -force simulations^16^ to reproduce the half-sarcomere force recovery phase following a rapid step in length (T2 curve^27–29^) with *model2*, the force–length relation of the Coulomb force that drives the lever arm is nearly compensated by parallel friction within the first $\approx \,0.1\,\hbox {ms}$ (Fig. 7^16^). Diminishing displacements within the Coulomb drive strongly suggests that the Coulomb contribution to $k_{hs}$ is, likewise, practically friction-neutralized at very high frequencies such as 4000 Hz. The latter seems to be supported by experimental data, since a half-sarcomere needs to elongate 4 nm to achieve a force enhancement of 180–200% at $F_{max}$ ($\approx \,3500\,\hbox {Hz}$), whereas an 8 nm elongation accompanies the same force at $100\,\hbox {Hz}$^38^. In fact, there have even been half-sarcomere stiffness estimations as low as $k_{hs}\,=\,10\,\hbox {pN}\,\hbox {nm}^{-1}$ for $<50\,\hbox {Hz}$^39^ and in slow ramp experiments^40^.

Although our MTC and CE stiffness fit courses shown in the supplementary Fig. S7 correlate well with other findings^19,20^, the exact number of formed cross-bridges is unknown to us. Piazzesi et al.^19^ also estimated that the maximum number of formed cross-bridges is $\approx \,90$ from single fibre experiments. If $n_{CB,max}\,=\,90$, then the force of a single cross-bridge is $\approx \,5\,\hbox {pN}$ ($\frac{445\,pN}{90}$), a value at which mechanical, structural, and energetic approaches seem to converge about (4–5 pN)^31,32^. The estimated $k_{hs}$ values for both models are robust towards the exact number (within limits) because for 90 formed cross-bridges, the stiffness of a single cross-bridge is $0.06\,\hbox {pN}\,\hbox {nm}^{-1}$ ($\frac{5\,\text {pN}}{85.7\,\text {nm}}$, *model1*) and $0.024\,\hbox {pN}\,\hbox {nm}^{-1}$ ($\frac{2.2\,{\text {pN}\,{nm}}^{-1}}{90}$, *model2*). Skeletal muscle structure and the principles of force generation are very similar in tetrapods ^41-43^. The sarcomere length^44^, the cross-bridge force of 4–5 pN^31,32^, filament stiffness^19^, as well as the muscle fibre material content in a whole muscle^45^ used in this study are estimations taken from various types of animals. These include rats^44^ and frogs^31,45^. Such combined sarcomere and cross-bridge values are often used as input or for validation of various general cross-bridge and half-sarcomere models, including both *model1*^15^ and *model2*^16^. Thus, we assume that our findings also apply to cross-bridge mechanics across various tetrapod species. However, a cross-bridge response will depend on the characteristic movements of each animal species (running, jumping with high impacts, or slow locomotion with low impacts).

In conclusion, we estimated the energy dissipated by the fibres (CE), and found that 0.6% of available mechanical work (per ATP) is dissipated by a cross-bridge at maximum isometric muscle force $F_{max}$ due to an impact. It is unlikely that the pre-activation required before touch-down generates $F_{max}$; instead a lower pre-activation, as found in humans, is more likely. Based on our data, we strongly think that the Wistar *m. gastrocnemius*, in-vivo and at intermediate running speed, dissipates by impacts about 16% of the mechanical work available throughout the period of hydrolysing one ATP molecule. Moreover, the GAS is such designed that the entire MTC is critically damped at TD due to submaximal pre-activation. Consequently, our new findings show that ignoring wobbling in muscle models, especially those emulating legged locomotion^10,11^, can lead to underestimating the energetic costs associated with walking or running. In addition to this, the energy dissipated due to wobbling is a vital piece of information when verifying muscle models that include visco-elastic properties such as *model2*^16^. Moreover, it seems there is no getting out of integrating representations of frictional mechanisms, next to muscle inertia, into explanatory models of highly dynamic muscle contraction.

Our scaled half-sarcomere stiffnesses are lower than compared to what has been found in slow ramp experiments for single fibres, and much lower than in rapid step-in-length and 4000 Hz oscillation fibre experiments. The majority of the stiffness difference can be explained by the actuating drive within a cross-bridge being caused by a Coulomb force that is friction-inhibited at very high frequencies, and subsequently by the possibility of local CE compression under an impact. In our experiments, we tried to emulate the impact that a rat would experience at an intermediate speed, which superimposed to the muscles a critically damped oscillation at roughly 60 Hz. It is unlikely that such high perturbations frequencies ($\approx \,4000\,\hbox {Hz}$), required to inhibit the suggested Coulomb-originating cross-bridge stiffness, can occur in legged locomotion.

## Materials and methods

### Ethics

We performed all experiments on five (N = 5) freshly killed rat (*Rattus norvegicus*, Wistar) muscles (*m. gastrocnemius medialis and lateralis*: GAS). These five GAS specimens were provided by another animal study that was approved according to Section 8 of the German animal protection law (Tierschutzgesetz, BGBl. I 1972, 1277; Reg.- Nr. 02-022/11; Thüringer Landesamt für Verbraucherschutz, Abteilung Gesundheitlicher und technischer Verbraucherschutz). This other study performed experiments on other leg muscles without impairing the GAS. They anaesthetised the rats with sodium pentobarbital (100 mg per 1 kg body mass), and the applicants of that study had no objection against GAS extraction immediately after the rats’ death. Anatomical data, specified as the mean of the five specimens, can be seen in Table 1.

### Whole muscle preparation and experimental procedure

**Figure 6:**
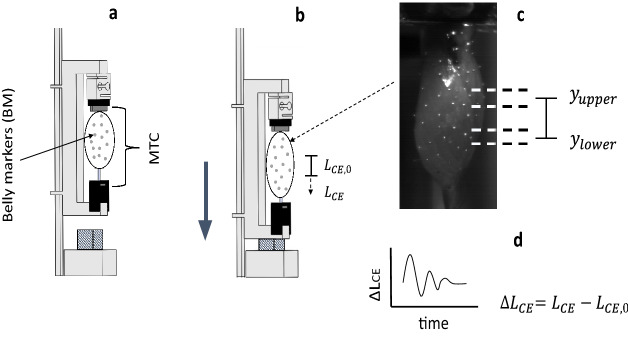
Drawing of the experimental setup. (**a**) The frame before TD. GAS is fixed between the upper and lower clamp (solid dark-grey rectangles). Above the upper clamp is an insulator (solid, black rectangle) and the force transducer, respectively, which are both fixated to the frame backbone (squared C-shape). The solid, black insulators prevent muscle stimulation to interfere with the force transducer. The light-grey spots on the muscle belly are illustrating the steel markers that pattern the muscle belly, which we used to calculate the dynamic force change between MTC ends in response to the impact ($\Delta F\,=\,\ddot{\overline{BM}}\cdot m\,=\,a_{COM}\cdot m$) after TD, with *m* being the GAS mass, $\overline{BM}$ the arithmetic mean of all belly markers’ vertical (*y*) positions and $a_{COM}$ the correspondingly estimated acceleration of the centre of mass. (**b**) The frame after TD with the polystyrene (hatched rectangle) being compressed. In (**b**), the belly’s stretch response to the impact is drawn exaggerated. (**c**) A video frame image of the muscle belly from one of the trial cameras, where the white spots are the steel markers, and the dashed, black lines are the upper and lower limits of the horizontally spread upper and lower ranges of CE markers for which $y_{upper}$ and $y_{lower}$, respectively, symbolise the arithmetic means of the vertical marker positions in each the upper and the lower range, with $L_{CE} = y_{upper} - y_{lower}$ and $L_{CE,0}$ the CE reference length fixed at TD. (**d**) an example of how $\Delta L_{CE}$ changes over time, after TD. A more detailed description of the functionality of the frame is given elsewhere^14^.

Once GAS was free from its surrounding tissues, except for small bone tissue pieces of the calcaneus and femur, the frontal surface of the muscle belly was patterned stochastically with high-grade steel markers (spheres, nominal diameter 0.4 mm, mensuration N0, IHSD-Klarmann, 96047 Bamberg, Germany). These steel markers were held in place by the adhesive surface of the CE in the same manner as the blunt bent wire that extended from the lower clamp. GAS was then vertically fixated between the upper and lower clamps that extruded from the cantilever arms of the frame (Fig. 6), with the bony tissue pieces of calcaneus and femur as fixation ‘clutches’.

MTC was stimulated (Aurora Scientific 701C) with $500\,\upmu \hbox {s}$ long square wave pulses of 10 V (three times the twitch threshold) at 100 Hz to ensure tetanic contraction during the trials, as recommended by a previous study^46^ . The stimulation in each trial lasted for 265 ms and was conducted with the GAS contracting isometrically at $L_{opt}$ while falling ($L_{opt}$ was inferred from^47^). Each series of falling experiments was finalised by a trial without stimulation, i.e., with passive muscle fibres. For preventing desiccation, the GAS surface was spray-moisturised after every second trial with Ringer’s solution. We performed all experiments at room temperature (23–25 $^{\circ }\hbox {C}$) within 60 min, to prevent irreversible tissue damage from lasting ischaemia conditions^17,18^.

## Data acquisition

We captured local muscle kinematics with two high-speed cameras (HCC-1000 BGE, VDS Vosskühler, 07646 Stadtroda, Germany), each of which recorded 256 $\times$ 1024 pixels per sample at 1825 Hz sampling rate. Both cameras were equipped with lenses of 25 mm focal length (Xenon 25/0.95, Schneider-Kreuznach, 55543 Bad Kreuznach, Germany) and custom-made 2 mm-extension tubes to minimise focusing distance, which gave a pixel resolution of $0.0064\,\hbox {mm}^2$. Sufficient light was provided by two stroboscopes (MultiLED PT, GSvitec GmbH, 63571 Gelnhausen, Germany).

### Data analysis

The data provided in the present paper have all not met the exclusion criteria (I)–(IV) given in Supplementary Text S1. The included data have been processed separately for each camera, whereafter we calculated the mean value between the two cameras. The reason for this was that not all markers were visible in one camera view despite a signal-to-noise ratio of 17.3 dB. The damping properties were highly sensitive to a potential one-sample ($0.5\,\hbox {ms}\,\approx \,\frac{1}{1825\,\text {Hz}}$) delay between the two cameras (Supplementary Text S2). We smoothed all our included raw data with a moving average filter with a kernel length of 5.

Since a very low portion of the mass was in the tendons, and GAS was suspended to a rigid construction, we estimated the MTC centre of mass (COM) with the kinematic information from all belly markers (arithmetic mean). Subsequently, we used the second derivative of COM ($a_{COM}$) to detect TD, i.e., the point in time when the frame made contact with the polystyrene (hatched square in Fig. 6). The $a_{COM}$ was further used to calculate the dynamic force change between MTC ends in response to the impact as $\Delta F\,=\,\hbox {GAS}\,\hbox {mass}\,\cdot \,a_{COM}$. The force transducer was only used to measure GAS isometric force just before TD, and TD was in each trial determined as the point before the earliest instant of $a_{COM}$ raised above the noise level^14^.

From marker kinematics, we segmented CE from MTC. The CE length ($L_{CE}$) was the vertical distance between two horizontal ranges that were located on solely fibre material; the vertical position of each range was calculated as the arithmetic mean of all markers it contained (see Fig. 6). From the $L_{CE}$ information, we calculated the CE elongation after TD: $\Delta L_{CE}\,=\,L_{ CE}-L_{CE,0}$, with $L_{CE,0}$ the CE reference length determined at TD. The optimal fibre length ($L_{opt}$) was defined as the measured GAS length with the knee and ankle joint at $90^{\circ }$ ( $L_{GAS,90^{\circ }}$), plus an added $2\,\hbox {mm}$ ($L_{GAS,90^{\circ }}\,+\,2\,mm\approx L_{opt}$), which was inferred from literature^47,48^. The reference length of the COM ($L_{COM,0}$) was the COM’s vertical distance to the frame marker at TD, and $\Delta L_{MTC}\,=\,L_{COM}-L_{COM,0}$ is the corresponding COM displacement after TD. The frame marker was located at the bony tissue piece of the calcaneus.

## Data interval

With known values for length (*L*(*t*)) and length rates ($\dot{L}(t)$), stiffness and damping properties were inferred from a 3-parameter function

1 $$\begin{aligned} F_i(L_i,\dot{L}_i)=k\cdot L_i+b+d\cdot \dot{L}_i , \end{aligned}$$

where *k* is the stiffness, *b* the intersection, and *d* the damping coefficient. The index *i* indicates the time samples of the analysed time period. This over-determined system of linear equations was solved for *k*, *b* and *d* by the Matlab operator “$\backslash$”. With this, we calculated $k_{CE}$, $k_{MTC}$, $d_{CE}$ and $d_{MTC}$.

Force-displacement data were analysed by using Eq. (1) in the time period between TD and when $a_{COM}$ returned to zero for the second time ($\approx$ 17 ms). Using the right Riemann summation method, we approximated the area enclosed by these work-loops for both MTC and CE.

## Scaling the contractile element (CE)

Under the assumption that the CE region is an isotropic and homogeneous material, we scaled the stiffness of the contractile element ($k_{CE}$) to the stiffness of a half-sarcomere $k_{hs}$ with

2 $$\begin{aligned} k_{hs} (k_{CE})=\frac{A_{hs}\cdot E}{L_{hs}}=\frac{A_{hs}\cdot \frac{k_{CE}\cdot L_{CE,0}}{r\cdot A_{CE,max,0}}}{L_{hs}} , \end{aligned}$$

where $L_{CE,0}$ and the maximum cross-sectional area ($A_{CE,max,0}$) are anatomical data from Table 1, and *E* is Young’s modulus. The half-sarcomere length ($L_{hs}$) is set to 1150 nm^44^, and the area of an elementary cell (1 myosin and 2 actin filaments) is $A_{hs}$ = 1540 nm$^{2}$ (Supplementary Fig. S6). For $A_{CE,max,0}$, we assumed that fibre material takes up 83% (*r* = 0.83)^45^ of a macroscopic muscle’s ACSA and that the remaining 17% does not carry any significant loads at these lengths^49,50^. The corresponding isometric force per half-sarcomere $F_{hs}$ was calculated as

3 $$\begin{aligned} F_{hs} (F)=\frac{F\cdot A_{hs}}{r \cdot A_{CE,max,0}} . \end{aligned}$$

We used the parameter $A_{CE,max,0}$ because our examined fibre area with $L_{CE,0}$ = 7.5 mm (Table 1) was located approximately at the muscle belly centre at which $A_{CE,max,0}$ applies, rather than $A_{CE,avr,0}$. *F* is the isometric force generated by the GAS MTC just before TD, which is measured by the force transducer.

By correspondingly applying the above scaling rules for lengths and forces, we estimated the work per half-sarcomere ($w_{hs}$, right axis Fig. 2c) as

4 $$\begin{aligned} w_{hs} (w_{CE})=\frac{ w_{CE}\cdot L_{hs}\cdot A_{hs}}{r \cdot L_{CE,0}\cdot A_{CE,max,0}} . \end{aligned}$$

The work of the contractile element ($w_{CE}$) was calculated as the area enclosed by a work-loop (see “Data interval”). The damping coefficient per half-sarcomere ($d_{hs}$) was calculated with

5 $$\begin{aligned} d_{hs}(d_{CE})\,=\frac{d_{CE}\cdot A_{hs}\cdot L_{CE,0}}{r\cdot A_{CE,max,0}\cdot L_{hs}} . \end{aligned}$$

The damping coefficient of the contractile element ($d_{CE}$) was inferred from Eq. (1). Comparing Eq. (2) to Eq. (5) reminds us that linear stiffnesses and damping coefficients scale the same with the dimensions of the finite volumes of which they represent these mechanical properties.

## Model ideas (short version)

In *model1*^15^ (Fig. 5), the half-sarcomere consists of two compartments *in-series*: the cross-bridges and the myofilaments. The force generated by a single cross-bridge is assumed to be a constant, with an associated constant deflection $\Delta L_{CB}$. The overall half-sarcomere force $F_{hs}$ equals the sum of all cross-bridge forces ($F_{CB}$), which scales linearly with the number of attached heads ($n_{CB}$), like the overall stiffness ($k_{CB}\,=\,\frac{F_{CB}}{\Delta L_{CB}}$) of the cross-bridge part. Knowing the constant parameters $\Delta L_{CB}$ and myofilament compliance $C_{fil}$, we can determine the half-sarcomere stiffness as

6 $$\begin{aligned} k_{hs}(F_{CB})=\frac{1}{C_{fil}+\frac{\Delta L_{CB}}{F_{CB}}} . \end{aligned}$$

In a fully fresh muscle with $F_{CB}\,<\,F_{CB,max}\,=\,445\,\hbox {pN}$, leaving both parameters in Eq. (6) open for a fit to the data in Fig. 4, we find $\Delta L_{CB}\,=\,85.7\,nm$ and $C_{fil}\,=\,0.4\,\hbox {nm}\,\hbox {pN}^{-1}$ (Table 2).

*model2*^16^ is more complex (Fig. 5): apart from the myofilaments, the cross-bridge itself is divided into a catalytic domain and a light chain domain that can rotate, actuated by a Coulomb force drive, with respect to the catalytic domain (both represent the S1 part). Combined, light chain, S2 part and the myofilaments form the (serial) elastic part, which we refer to by the stiffness symbol $k_{fil}$ further below. The underlying model idea consists of a repulsing Coulomb force generated within the catalytic domain, which upon myosin head attachment causes a driving force acting between the catalytic and the light chain domains. The driving force then levers the light chain such that the cross-bridge can generate force between the actin and the myosin filaments (Fig. 2^16^). According to *model2*, the force ($F_{CB}(L_{CB})$) generated by the attached cross-bridges in a half-sarcomere is a non-linear function of the model-internal lever arm coordinate $L_{CB}$ (Fig. 2^16^), and the corresponding cross-bridge stiffness ($k_{CB}$) is

7 $$\begin{aligned} k_{CB} (F_{CB})=2\cdot F_{CB,max}\cdot \sqrt{\frac{\left( \frac{F_{CB}}{F_{CB,max}}-\frac{-c_1}{c_{3}^2}\right) ^3}{c_1}} , \end{aligned}$$

where $F_{CB,max}$ is the maximum force generated by the cross-bridge ensemble in a half-sarcomere (their current number: $n_{CB}$). The $c_{1}$ is a constant that depends on $c_{3}$ and assumes the lever coordinate $L_{CB,opt}$ is at its optimal lever arm position corresponding to a cross-bridge generating about $F_{CB,1}=\,4\hbox {-}5\,\hbox {pN}$ ($F_{CB,max} = n_{CB,max} \cdot F_{CB,1}$, with $n_{CB,max} \approx 90$; for more detail regarding Eq. (7), see Supplementary Text S3). At $L_{CB}\,=\,-c_{3}$ the assumed function $F_{CB}(L_{CB})$ of the cross-bridge-internal force–length relation has a pole.

In line with *model1*, $k_{CB}$ in *model2* acts *in-series* with myofilament (plus S1) stiffness $k_{fil}\,=\,\frac{1}{C_{fil}}\,=\,150\,\hbox {pN}\,\hbox {nm}^{-1}$^19^ to make up an overall $k_{hs}$. Further, we assumed that all cross-bridges in *model2* are always at $L_{CB,opt}\,=\,7\,\hbox {nm}$. With this, just like in *model1*, both the isometric force $F_{CB}\,=\,u \cdot F_{CB,max}$ and the cross-bridge stiffness $k_{CB}\,=\,u \cdot k_{CB,max}$ are assumed to scale linearly solely with the number $n_{CB}$ of attached myosin heads ($u\,=\,\frac{n_{CB}}{n_{CB,max}}$). Thus, the overall $k_{hs}$ for *model2*, when additionally using the latter assumption, can then be expressed as

8 $$\begin{aligned} k_{hs}(u)=\frac{u\cdot k_{CB,max}\cdot k_{fil}}{u\cdot k_{CB,max}+k_{fil}} , \end{aligned}$$

Accordingly, this leaves only the $c_{3}$ value open for fitting (see Table 2) in Eq. (7) because $k_{CB,max}\,=\,k_{CB} (F_{CB}\,=\,F_{CB,max})$. Both model ideas, *model1* and *model2*, were fitted (see Fig. 4) with Matlab *cftool* (curve fitting tool).

## Supplementary Information

Supplementary Information.
