# Supplementary material for: Cross-bridge mechanics estimated from skeletal muscles’ work-loop responses to impacts in legged locomotion
Source: Sci Rep. 2021 Dec 8;11:23638. doi: 10.1038/s41598-021-02819-6 (PMC8655009; doi:10.1038/s41598-021-02819-6)
Supplement: Supplementary file 1 — Supplementary Information. [file 41598_2021_2819_MOESM1_ESM.pdf]

Supplementary Information for

Cross-bridge mechanics estimated from skeletal muscles' work-loop responses to impacts in legged locomotion

Kasper B. Christensen, Michael Günther, Syn Schmitt and Tobias Siebert

This file includes:
Supplementary Text S1-7
Supplementary Table S1-3
Supplementary Figure S1-7
References

Supplementary Text S1

Exclusion criteria

Not all data were suitable for use within our analyses; therefore, we used exclusion criteria at different stages of data processing. In particular, we excluded the following: (I) an entire trial if the force at TD was less than 95% of the trial-specific isometric force measured just before or after the impact response; (II) an entire trial if material shortening ($\epsilon_{CE} < 0$) preceded material elongation as an initial response to TD; (III) a marker in a trial if it had glided obviously across the muscle surface during the experiment; and (IV) a marker in a trial if it showed phase and/or amplitude irregularities (either in coordinate position or acceleration) when compared to all other markers.

Supplementary Text S2

Dealing with the non-synchronicity of cameras and the critical choice of the marker on the frame

To calculate work loops and damping coefficients in MTC and CE in response to impacts, we had to remove two potential sources of systematic errors in the data analysis. Supplementary Fig. S1 shows the acceleration signals of two different marker positions on the frame (*hook* and *insulator*). The *hook* marker was on the aluminium part of the lower clamp, formed as a hook, and the *insulator* marker was on the outside of the left, lower insulator (see [Fig. 1] [1]). In general, while the *hook* marker had a higher noise-to-signal ratio than the *insulator* marker, the *hook* systematically led the *insulator* trajectory by 0.5 ms. The latter finding is decisive for the calculation of reliable work-loops. The influence of the frame marker position used to calculate ΔL_{MTC} with and without a 0.5 ms frame marker delay are shown as examples in Supplementary Fig. S2b and a, respectively. Accordingly, the MTC was found to dissipate 45.6 μJ during one full oscillation period after TD if *hook* was used to calculate ΔL_{MTC} (no marker delay), whereas only 2.7 μJ would be calculated if *insulator* was used to calculate ΔL_{MTC} (marker delay). The frame marker used to calculate ΔL_{MTC} should ideally not even be on the lower clamp, rather, on the bony tissue of calcaneus or femur to guarantee the elimination of any potential frame property interference. In these experiments, however, both bony tissues proved unreliable for marker tracking.

The VDS Vosskühler cameras used in these experiments had an internal hardware buffer (ring buffer), thus, when the cameras were ready to record, waiting for an external hardware trigger, images started to fill the internal camera buffer memory (continuously removing the earliest entry when full). The ring buffer then potentially may or may not add a 0.5 ms ($\frac{1}{1825}$ Hz) offset to one of the two cameras, when the hardware trigger impulse enters. This potential recording discrepancy made the analysis susceptible to a similar effect as the frame marker delay above. In Supplementary Fig. S2c (same trial as above),

no camera offset (uncorrected data) yielded a negative energy dissipation of -14.6 μJ for the MTC as a mean of both cameras. On the contrary, a manually enforced 1-frame delay (0.5 ms) to one of the two cameras resulted in a mean MTC energy dissipation of 51.5 μJ (Supplementary Fig. S2d) during one full oscillation period after TD.

Nonetheless, some calculated mechanical properties, such as strains and stiffnesses of the MTC and CE are robust with regard to camera synchronisation and marker position delay. In the four cases considered above, the estimated MTC stiffness values, from a 3-parameter fit (Eq. 1, main text), span between 1560 N m^{-1}, 1630 N m^{-1}, 1620 N m^{-1}, and 1530 N m^{-1} for Supplementary Fig. S2a,b,c and d, respectively.

To systematically check for camera offsets, we used three different methods of detection (Supplementary Table S1). It was common for all three methods that we tried to detect an equal number of steel markers in each of the two cameras. In the first method, we calculated the arithmetic mean of all individual tracked muscle belly frame numbers, at which the markers reached their lowest vertical positions, and subsequently subtracted and rounded the difference to the nearest integer (*MinPos*). In the second method, the TD frames were found in each camera and then subtracted (*ImpIdx*). Thirdly, we compared the frame number that included the lowest vertical acceleration (a_{COM}) value in each camera (*MinAcc*), which we chose due to its narrow and easily detectable minimum. All three methods indicated a camera offset of either 1, 0, or -1. As seen in Supplementary Table S1, there were camera offset indications throughout the experiments, but all indicators proved to be inconsistent. As a consequence thereof, we chose to do all work-loop calculations separately for each camera, and present our results for each trial as the mean value of the data from the two cameras.

Supplementary Text S3

Model ideas(extended)

model1 [2]

According to *model1* (Eq. 1), all cross-bridges act *in parallel*, and they are assumed to be arranged *in series* with the myofilaments (actin and myosin), to make up the half-sarcomere stiffness

$$k_{hs}(F_{CB}) = \frac{1}{C_{fil} + \frac{\Delta L_{CB}}{F_{CB}}} \quad . \tag{1}$$

Here, $C_{fil} = \frac{1}{k_{fil}}$ is the overall compliance of the myofilaments (k_{fil} their stiffness) and $\frac{\Delta L_{CB}}{F_{CB}} = \frac{1}{k_{CB}}$ the overall compliance of the ensemble of n_{CB} cross-bridges (k_{CB} their stiffness) in a half-sarcomere.

The force generated by a single cross-bridge ($F_{CB,1}$) is assumed to be a constant, with an associated constant deflection (ΔL_{CB}). The overall stiffness

of the cross-bridge ensemble, which generates the half-sarcomere force

$$F_{hs}(n_{CB}) = F_{CB}(n_{CB}) = n_{CB} \cdot F_{CB,1} \quad , \tag{2}$$

scales linearly, just like the force, with the number of cross-bridges:

$$k_{CB}(n_{CB}) = \frac{F_{CB}}{\Delta L_{CB}} = n_{CB} \cdot k_{CB,1} \quad , \tag{3}$$

where $k_{CB,1} = \frac{F_{CB,i}}{\Delta L_{CB}}$ is the stiffness of a single cross-bridge. With known values for k_{fil} and n_{CB}, the overall half-sarcomere stiffness (Eq. 1) can also be written as

$$k_{hs}(n_{CB}) = \frac{k_{CB}(n_{CB}) \cdot k_{fil}}{k_{CB}(n_{CB}) + k_{fil}} \quad . \tag{4}$$

Since ΔL_{CB} of each cross-bridge is fixed at $\Delta L_{CB} = 1.5\,\mathrm{nm}$ [2], and cross-bridges and filaments are arranged *in series*, the half-sarcomere elongation is

$$\Delta L_{hs}(n_{CB}) = \Delta L_{CB} + \Delta L_{fil}(n_{CB}) \quad , \tag{5}$$

with the filament elongation being

$$\Delta L_{fil}(n_{CB}) = \Delta L_{CB} \cdot \frac{k_{CB}(n_{CB})}{k_{fil}} \quad . \tag{6}$$

In accordance with *model1*, the cross-bridge force (F_{CB}) is transmitted by the filament ($F_{CB} = F_{hs} = F_{fil}$); thus, we can write

$$F_{CB}(n_{CB}) = k_{hs}(n_{CB}) \cdot \left(\Delta L_{CB} + \Delta L_{fil}(n_{CB})\right) \quad . \tag{7}$$

To reproduce [Fig. 3 A,B,C] [2], we calculated k_{hs} from Eq. 4—and further using Eq. 3— with k_{fil} values of either $150\,\mathrm{pN\,nm^{-1}}$ and $k_{CB,1} = 3\,\mathrm{pN\,nm^{-1}}$ (solid, black line in Supplementary Fig. S3) or $k_{fil} = 90\,\mathrm{pN\,nm^{-1}}$ and $k_{CB,1} = 1\,\mathrm{pN\,nm^{-1}}$ (dashed, black line in Supplementary Fig. S3); for this, both k_{hs} variations assume $\Delta L_{CB} = 1.5\,\mathrm{nm}$ [2], thus, $F_{CB,1} = 4.5\,\mathrm{pN}$ or $1.5\,\mathrm{pN}$, respectively. For all four graphs in Supplementary Fig. S3 (including *model2* and an alternative *model2* version), we assumed that the maximally possible number of cross-bridges in a half-sarcomere sub-unit (i.e. one half-myosin and two actin filaments) is $n_{CB} = n_{CB,max} = 90$ [3].

model2 [4]

The overall idea for *model2* is that a half-sarcomere consists of an active element (AE), a parallel damping element, a serial elastic element and a serial damping element. In particular, the cross-bridge itself is divided into a catalytic domain and a light chain domain that can rotate, assumed to be actuated by a Coulomb force drive (the AE) with respect to the catalytic domain (together, they represent the S1 part of a myosin molecule). The levered light chain, in turn, generates force (F_{AE}) between the actin and the myosin filaments, which is a non-linear function of the internal lever arm coordinate (L_{AE}) [4]. The serial

elastic element in the model includes all parts other than AE (like S1, S2, filaments) in series to the rotational degree of freedom constituting the AE.

To compare *model1* and *model2*, we only included the AE and the serial elastic element of *model2*—leaving out the dampers. We further assumed that the AE is the same as the cross-bridge in *model1* (AE $\widehat{=}$ CB) and that the serial elastic element of *model2* corresponds to the filament stiffness in *model1* (k_{fil}). With this, the sum force

$$F_{CB}(L_{CB}) = F_{CB,max} \cdot \left(\frac{c_1}{(L_{CB} + c_3)^2} + c_2 \right) \tag{8}$$

of all cross-bridges in a *model2*-like half-sarcomere is a non-linear function of the L_{CB} position of the representative cross-bridge. In Eq. 8, c_1, c_2, c_3 are parameters and $F_{CB,max}$ is the maximal force that the cross-bridge drive of a half-sarcomere can generate at a corresponding optimal lever coordinate ($L_{CB} = L_{CB,opt}$ see Supplementary Fig. S5); c_1 can be written in terms of c_3 and the optimal cross-bridge lever coordinate:

$$c_1 = \frac{{c_3}^2 \cdot (L_{CB,opt} + c_3)^2}{{c_3}^2 - (L_{CB,opt} + c_3)^2} \quad ; \tag{9}$$

c_2 (> 1) can be written in terms of c_1 (< 0) and c_3 (> 0):

$$c_2 = \frac{-c_1}{{c_3}^2} \quad ; \tag{10}$$

c_3 fixes the pole coordinate for the model function $F_{CB}(L_{CB})$ where F_{CB} approaches infinity ($L_{CB} = -c_3$).

For Eq. 8, the length-derivative of F_{CB}, i.e. k_{CB}, can be expressed as

$$k_{CB} = F'_{CB} = \frac{d}{dL_{CB}} \cdot \left(F_{CB,max} \cdot \left(\frac{c_1}{(L_{CB} + c_3)^2} + c_2 \right) \right) \tag{11}$$

$$= F_{CB,max} \cdot c_1 \cdot \frac{d}{dL_{CB}} \cdot \left((L_{CB} + c_3)^{-2} \right) \quad . \tag{12}$$

Eq. 12, we can express the stiffness of the cross-bridges' part as a function of L_{CB}:

$$k_{CB}(L_{CB}) = -\frac{2 \cdot c_1 \cdot F_{CB,max}}{(L_{CB} + c_3)^3} \quad . \tag{13}$$

To enable an immediate comparison with the half-sarcomere stiffness predicted by *model1*, we now replace—by solving Eq. 8 for $(L_{CB} + c_3)^2$ and inserting this

into Eq. 13—the variable length L_{CB} by the force F_{CE} in Eq. 13, and find

$$
\begin{aligned}
k_{CB}(F_{CB}) &= -\frac{2 \cdot c_1 \cdot F_{CB,max}}{\left(\frac{c_1}{\frac{F_{CB}}{F_{CB,max}} - c_2}\right)^{\frac{3}{2}}} & (14)\\
&= -2 \cdot c_1 \cdot F_{CB,max} \cdot \left(\frac{\frac{F_{CB}}{F_{CB,max}} - c_2}{c_1}\right)^{\frac{3}{2}} & (15)\\
&= \frac{2 \cdot c_2 \cdot F_{CB,max}}{c_3} \cdot \left(1 - \frac{F_{CB}}{c_2 \cdot F_{CB,max}}\right)^{\frac{3}{2}} \quad , & (16)
\end{aligned}
$$

with the last form (Eq. 16) resulting from substituting $c_1 = -c_2 \cdot c_3^2$ (according to Eq. 10) into Eq. 15 and eventually extracting c_3 and, as much as possible, c_2 from the $(\ldots)^{\frac{3}{2}}$-term. For the values $L_{CB,opt} = 7\,\text{nm}$ and $c_3 = 1.2$ (Table 2) to $4\,\text{nm}$ [4], which are characteristics for a cross-bridge, the values of parameter c_2 are close to unity: $c_2 = 1.02$ to 1.15 [4].

Both *model2* and *model1* assume that the stiffnesses of the cross-bridge and filament parts arranged *in series* make up the overall half-sarcomere stiffness. Also in *model2*, just like in *model1*, the isometric force F_{CB} (of a half-sarcomere: $F_{CB} = F_{hs}$) scales linearly with the number of cross-bridges (in a half-sacomere: n_{CB}), which are all acting at the optimal lever coordinate $L_{CE,opt}$:

$$
F_{hs}(n_{CB}) = F_{hs}(u) = u \cdot F_{CB,max} \quad , \tag{17}
$$

with $u = \frac{n_{CB}}{n_{CB,max}}$ being a normalised factor. Therefore, the stiffness of the cross-bridge part scales likewise linearly with u, and the stiffness of the serial arrangement in the half-sarcomere becomes

$$
k_{hs}(n_{CB}) = k_{hs}(u) = \frac{u \cdot k_{CB,max} \cdot k_{fil}}{u \cdot k_{CB,max} + k_{fil}} \quad , \tag{18}
$$

with $k_{CB,max} = k_{CB}(F_{CB,max})$ (Eq. 16). In Supplementary Fig. S3, Eq. 18 is used to calculate k_{hs} with $n_{CB} = n_{CB,max} = 90$, $L_{CB,opt} = 7\,\text{nm}$ and $k_{fil} = 150\,\text{pN}\,\text{nm}^{-1}$. An alternative version of *model2* (all attached) is also plotted in Supplementary Fig. S3, where the term $u \cdot k_{CB,max}$ in Eq. 18 is substituted with Eq. 13. Thus, the force per half-sarcomere no longer scales with the number of cross-bridges but with the lever arm coordinate of the representative cross-bridge: all myosin heads are assumed to be always attached ($u = 1$: $n_{CB} = n_{CB,max}$) and deflected in their work-stroke so as to generate exactly the force $F_{CB} \leq F_{CB,max}$ demanded.

Supplementary Text S4

A $F_{hs,max}$ comparison to literature

In this paper, the parameters characterising particularly two basic structural properties were taken from literature: The physiological cross-sectional area

(PCSA) of the muscle consists of 83 % myofibril material [5], and the elementary cell of the cross-sectional filament lattice in sarcomeres has the shape of a parallelogram with an area of 1540 nm^2 (Supplementary Fig. S6) at optimal half-sarcomere length (1150 nm). With our measured maximum muscle stress (240 $kN\,m^{-2}$) value, the corresponding $F_{hs,max} = 445$ pN value for a half-sarcomere can then be estimated by use of the two parameter values just given above.

To estimate the uncertainty of our predicted $F_{hs,max}$ value, it may be assumed that only 80 % [6,7] of the PCSA is myofibril material, and that the interplanar d(1,0) lattice distance is 40 nm^2 [6] (the shape of the elementary cell is either calculated as a square or a parallelogram). In Supplementary Table S2, a summary of the parameter variations given above with data on maximum isometric muscle stress likewise taken from literature, e.g. from two prominent papers [3,8]. Accordingly, since our measured muscle stress (240 $kN\,m^{-2}$) value is identical to Piazzesi *et al.* (2007), the percentage variance of $F_{hs,max}$ is about 25 % ($\frac{554\,\text{pN} - 445\,\text{pN}}{445\,\text{pN}}$), depending on the parameters used for the $F_{hs,max}$ calculation (Supplementary Table S3).

Supplementary Text S5

Falling height (experimental setup)

As compared to our previous experiments [1], the falling height of the customised frame (Fig. 6) was reduced from 4 cm to 1 cm to impose only half the impact force. The relation between falling height (h) and the mean (ground reaction) force on the frame during the impact (ΔF_{imp}) arises from combining the conservation of mechanical energy (Eq. 19) and the impulse-momentum equation (Eq. 20) (for detailed information of frame and previous experimental setup, see [1]),

$$mgh = \frac{1}{2}mv^2 \rightarrow v = \sqrt{2gh} \tag{19}$$

and

$$p = mv = \Delta p \rightarrow \Delta F_{imp} = \frac{\Delta p}{\Delta t} = \frac{m\sqrt{2gh}}{\Delta t} \quad , \tag{20}$$

respectively, with m being the frame mass (52 g, twenty-six times higher than MTC mass: Table 1 , v the frame's TD velocity, p the frame's linear momentum at TD, Δp its change (impulse) during the impact of duration Δt and g the gravitational acceleration.

Supplementary Text S6

Fatigue

In our experiments, the average time between each trial was 254 seconds, with the first measurements conducted $t \approx 10$ minutes after dissection (t=0). Due to the *ex-vivo* experimental setup, the GAS' measured isometric force declined trial by trial (Fig. 1). The slope of the observed isometric force decline, solid line:

$2.9{\cdot}10^{-4}\ F_{max}\ \mathrm{s}^{-1}$ in Fig. 1, is almost identical to previously found data in similar experiments [1] However, a difference between previous and present experiments was the average time between each stimulation: here, we stimulated GAS once per 254 seconds, as compared to every 428 seconds in our previous experiments [1].

The muscles were on average stimulated once per 254 seconds for 265 ms, which is roughly half the stimulation interval from what has previously reported in a similar study (once per 428 seconds for 265 ms) [1]. Therefore, ischaemia seems so dominant for GAS isometric force production, that a reduction in stimulation duration had little to no influence on isometric force decline over the course of the experiments. The $F_{max} = 23\,\mathrm{N}$, which is lower than previously found (30 N [1]), can be explained by the difference in maximum belly anatomical cross-sectional area in both experiments (here, $A_{CE,max,0} = 96\,\mathrm{mm}^2$, Table 1). Since the maximum isometric tension in a muscle is between $2.5{\cdot}10^5\ \mathrm{N\,m}^{-2}$ [9] and $3 \cdot 10^5\ \mathrm{N\,m}^{-2}$ [10], and GAS has a pennation angle of 14° under similar isometric conditions [11], the expected F_{max} value is between 21 N and 28 N.

Supplementary Text S7

Titin and passive strain relationship

In our experiments with passive muscles, the mean initial GAS force measured by the force transducer was 0.25 N, with an added ΔF peak of 0.2 N as a response to the impact. Therefore, we estimate that the initial passive force and the force change for a half-sarcomere as a response to the impact were 4.8 pN and 3.9 pN (Eq. 3, main text), respectively. The median passive strain peak with ΔF was about 0.8%, which corresponds to an about 10 nm stretch peak per half-sarcomere (titin filament). Under the assumption that six titin filaments are attached to one myosin rod [12,13], and the slope of the force-length relation of a single titin filament, measured during fixed-rate stretches, is $0.05\,\mathrm{pN\,nm}^{-1}$ [14] between 0.8 pN and 1.5 pN, then the overall six-titin slope in a passive half-sarcomere would be $0.3\,\mathrm{pN\,nm}^{-1}$. The single-titin slope was estimated at $1000\,\mathrm{nm\,s}^{-1}$ stretch rate [14], which is practically the same as our rates measured for a half-sarcomere ($\approx 1100\,nm\,s^{-1}$), as the median stiffness in our passive experiments is $0.4\,\mathrm{pN\,nm}^{-1}$, the force change is 3.9 pN, and the time that passed between TD and this force change is 9 ms (Supplementary Fig. S1).

To understand how titin might influence the passive macro CE, we scaled the estimated titin slope to one specific passive trial: the CE work-loop in Supplementary Fig. S7 (dashed, light-grey line). We examined the scaled titin force-length relation while hypothesising two different, somehow extreme, conditions that may determine the work-loop return path from maximum elongation ($\Delta F > 0$) back to $\Delta F = 0$. As ΔF returns to zero, the titin filament would, in the respective two extreme cases, either shorten along our measured ΔL_{CE} path (Supplementary Fig. S7, solid, light-grey line), or along the same $0.3\,\mathrm{pN\,nm}^{-1}$ titin slope that applies to the stretch path (Supplementary Fig. S7, dotted, light-grey line). If titin re-shortens along the measured ΔL_{CE} path, then this would resemble a course of titin shortening after stretches at least an order of magnitude [14] longer than estimated in our trials. In such a case, titin accounts for

half (1.8 zJ, hatched area in Supplementary Fig. S7) of the 3.5 zJ dissipated CE energy for $\Delta F > 0$ in Supplementary Fig. S7.

On the other hand, Ig and PEVK unfolding events has been shown to occur above 6 pN [14,15], which is far greater than the estimated $\frac{3.9\,\mathrm{pN}}{6} = 0.65\,\mathrm{pN}$ force change in our experiments. If the unfolding events are the cause for a titin's viscosity, then titin might respond as a nearly non-dissipative (conservative) material within our given (low) force range. For the latter extreme case, titin would account for 84% of the 2870 $\mathrm{N\,m^{-1}}$ passive CE stiffness value in the Supplementary Fig. S7 example and 75% of the 3200 $\mathrm{N\,m^{-1}}$ median passive CE stiffness value, both estimated from a 3-parameter fit (Eq. 1, main text).

Titin is a visco-elastic material [14,15], and titin's ability to dissipate energy does not only depend on velocity. Titin's energy dissipation also depends on contractile history and time between each stretch/shortening contraction [14,16]. Furthermore, the resistance of passive muscle to movement (and thus its stiffness) increases with time at rest and is reduced by movement [17]. Thus, we suspect that the large deviations in energy dissipated in the passive trials might relate to either the time between the last active trial and the passive one, the time between dissection and the passive trial, or the total number of trials for a specific muscle. However, our sample size is too small, and our data too few to further explore any of these potential correlations.

	**max dev**	**0 dev**	**1 dev**	**2 dev**	**3 dev**	**N**
**MinPos**	1	20	20	-	-	40
**ImpIdx**	3	18	16	4	2	40
**MinAcc**	1	24	20	-	-	40
**MinPos|ImpIdx***	3	18	17	4	1	40
**MinPos|MinAcc ***	1	26	14	-	-	40
**ImpIdx|MinAcc***	3	17	20	2	1	40
**AllSame***	-	12	-	-	-	

Supplementary Table S1 | Camera recording inconsistencies within each trial that did not fulfil the exclusion criteria. The table lists the three methods of detecting inconsistencies (*MinPos*, *ImpIdx*, *MinAcc*) and the consistency between them (**MinPos**|**ImpIdx**, **MinPos**|**MinAcc**, **ImpIdx**|**MinAcc**). **AllSame** is number of trials where all three methods of detecting (**MinPos**, **ImpIdx**, **MinAcc**) are consistent. All three methods and comparisons of methods, are listed with the maximal found frame deviation(**max dev**) and how the found deviations, e.g. the number of frames, are distributed (**0 dev, 1 dev, 2 dev, or 3 dev**) across all trials (N). For the three methods, the **MinPos**, is the difference between the lowest centre of mass (COM) position in each of the two cameras. **ImpIdx**, is the frame difference between the touch-down (TD) index value in each of the two cameras. **MinAcc**, is the frame difference between the lowest acceleration of COM (a_{COM}) value in each of the two cameras.

* The numbers given here compares the inconsistencies between thw methods of detection, i.e. how often two methods has the same findings for a trial.

Data	Symbol	Unit	Source
240	σ	$\mathrm{kN\,m^{-2}}$	text [3]
206	σ	$\mathrm{kN\,m^{-2}}$	[table 2] [8]
40	d(1,0)	nm	d(1,0) = 40 nm [6]
0.8	r	a	80% of PCSA are myofibrils [6,7]
0.83	r	a	83% of PCSA are myofibrils [5]
1600	A	$\mathrm{nm^2}$	$d(1,0)^2$
1848	A	$\mathrm{nm^2}$	$d(1,0)^2 \cdot 2 \cdot \frac{1}{\sqrt{3}}$

Supplementary Table S2 | Literature data to infer F_{hs}. The two stress values from literature were used to infer force ($F = \sigma \cdot A$ or $F = \sigma \cdot A \cdot \frac{1}{r}$) for a representative half-sarcomere. The length values of the elementary cell's interplanar d(1,0) lattice distance in a half-sarcomere and the myofibril density (see Supplementary Fig. S6) are from independent literature sources. The area of the elementary cell was then either calculated as a square or a parallelogram.

Piazzesi *et al.* (2007) [3]	Lombardi *et al.* (1992) [8]
$240\,\frac{\mathrm{kN}}{\mathrm{m}^2} \cdot 1540\,\mathrm{nm}^2 \cdot 10^{-3} \cdot \frac{1}{0.83} = 445\,\mathrm{pN}$	$206\,\frac{\mathrm{kN}}{\mathrm{m}^2} \cdot 1540\,\mathrm{nm}^2 \cdot 10^{-3} \cdot \frac{1}{0.83} = 382\,\mathrm{pN}$
$240\,\frac{\mathrm{kN}}{\mathrm{m}^2} \cdot 1600\,\mathrm{nm}^2 \cdot 10^{-3} \cdot \frac{1}{0.8} = 480\,\mathrm{pN}$	$206\,\frac{\mathrm{kN}}{\mathrm{m}^2} \cdot 1600\,\mathrm{nm}^2 \cdot 10^{-3} \cdot \frac{1}{0.8} = 412\,\mathrm{pN}$
$240\,\frac{\mathrm{kN}}{\mathrm{m}^2} \cdot 1848\,\mathrm{nm}^2 \cdot 10^{-3} \cdot \frac{1}{0.8} = 554\,\mathrm{pN}$	$206\,\frac{\mathrm{kN}}{\mathrm{m}^2} \cdot 1848\,\mathrm{nm}^2 \cdot 10^{-3} \cdot \frac{1}{0.8} = 476\,\mathrm{pN}$

Supplementary Table S3 | $F_{hs,max}$ estimations. The variations in estimated $F_{hs,max}$ in two papers using literature data from Supplementary Table S2.

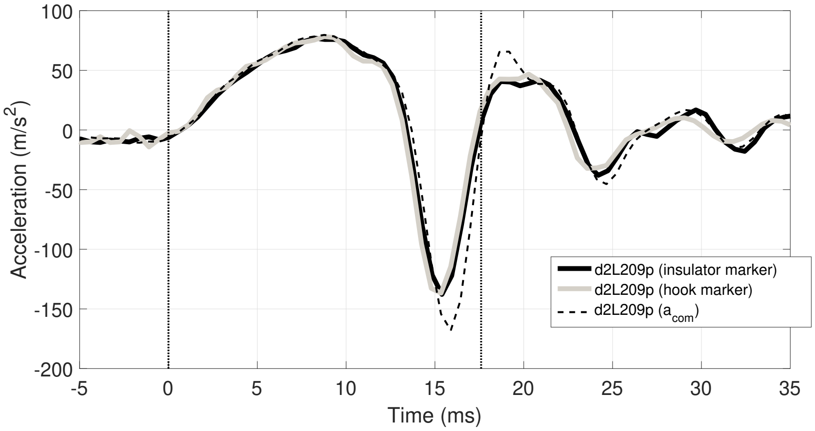

Supplementary Figure S 1 | Example of noise and delay in frame marker acceleration before and after TD. The solid, black line is the acceleration of the frame marker located at the left, lower insulator. Solid, grey line is the acceleration of the frame marker located on the lower clamp. The solid, grey line leads solid, black line with 0.5 ms (1 frame) in d2L209p. This trailing tendency was examined in 23 trials, and at no point was *insulator* earlier than *hook*. In addition, the solid, grey line never led with more than 0.5 ms. In few trials no delay was found. The dashed, black line is a_{COM} signal for this trial and the dotted, vertical, black lines indicate the span between TD and when a_{COM} returns to zero for the second time.

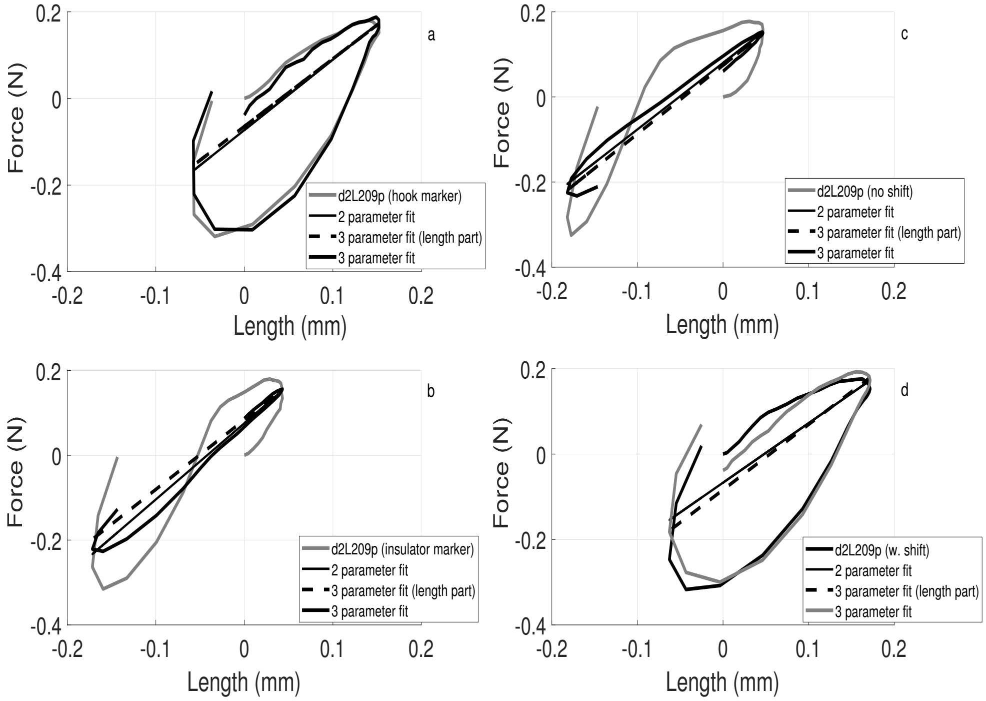

Supplementary Figure S2 | An example of time delays effecting the work-loop in trial d2L209p. **a** The *hook* marker, which had a systematic 0.5 ms lead on the *insulator* maker (Supplementary Fig. S1), was used to calculate ΔL_{MTC}, based on one camera view. **b** *insulator* used to calculate ΔL_{MTC}, based on the same view. **c**, d2L209p with the one frame shift between the two cameras as determined by MinPos (Supplementary Table S1). **d** d2L209p with a one frame enforced shift between the two cameras, ignoring the time delay determined by MinPos. In **a,b,c,d**, the solid, grey line is the measured ΔL_{MTC},ΔF response (work-loop) to the impact. The solid, thin, black line is the respective linear 2-parameter (force (length)) fit to the data. The solid, black loops depict the respective 3-parameter fits to the data, using the parameters k_i, b_i, d_i of each the function $F_i(L_i, \dot{L}_i)$ (see Eq. 1, main text), which linearly depends on length L_i and time rate of length change $\dot{L}_i$; the dashed, black line is the respective length-dependent contribution. d2L209p work-loop encompasses one oscillation period that spans between TD and the instant closest to zero when a_{COM} returns to zero for the second time (Supplementary Fig. S1).**a** is the same figure as Fig. 3b.

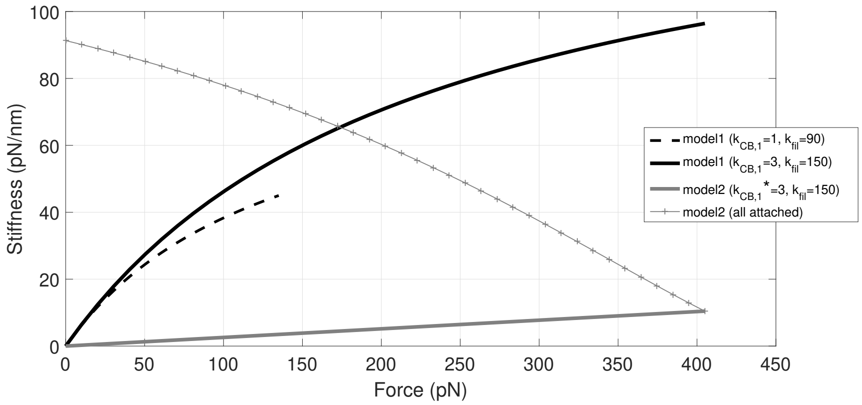

Supplementary Figure S3 | Model predicted stiffnesses as function of half-sarcomere force. The solid, black line is the predicted half-sarcomere stiffness k_{hs} for *model1* if $k_{CB,1} = 3\,\mathrm{pN\,nm^{-1}}$ (stiffness of a single cross-bridge), $k_{fil} = 150\,\mathrm{pN\,nm^{-1}}$ (filament stiffness) and a constant $\Delta L_{CB} = 1.5\,\mathrm{nm}$. The dashed, black line is the predicted k_{hs} for *model1* with $k_{CB,1} = 1\,\mathrm{pN\,nm^{-1}}$, $k_{fil} = 90\,\mathrm{pN\,nm^{-1}}$ and $\Delta L_{CB} = 1.5\,\mathrm{nm}$. The solid, grey line is the predicted *model2* k_{hs} if parameter values are the same (except $F_{CB,opt,1} = 4.5\,\mathrm{pN}$) as in the original paper for the non-linear $F_{CB}(L_{CB})$ ($k_{CB,1}{*} = 3\,\mathrm{pN\,nm^{-1}}$, $k_{fil} = 150\,\mathrm{pN\,nm^{-1}}$, $L_{CB,opt} = 7\,\mathrm{nm}$, $c_3 = 4\,\mathrm{nm}$), and force is proportional to number of cross-bridges, with the latter all at their optimal lever coordinate ($L_{CB,opt}$). The solid, thin, plus-sign, grey line is k_{hs} predicted from *model2* if the force changes in work-stroke with the (representative) lever coordinate instead of the number of attached cross-bridges ($n_{CB,max}$ myosin heads attached). For all calculations, we assumed $n_{CB,max} = 90$ [3] as the maximum number of cross-bridges.

* $k_{CB,1}$ as in Eq. 3 and subsequently used to estimate F_{CB} in Eq. 7, which is equal to F_{CB} in Eq. 13 that is used to calculate k_{CB} for *model2*.

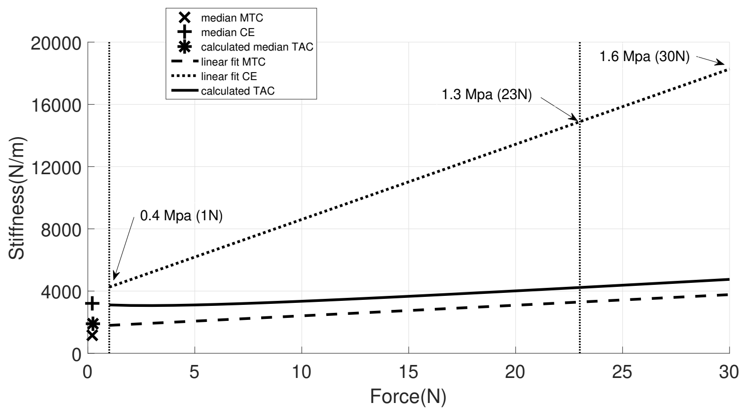

Supplementary Figure S4 | TAC stiffness (k_{TAC}) from linearly interpolated stiffnesses of CE (k_{CE}) and MTC (k_{MTC}) at TD. This figure shows MTC subdivided into a tendon-aponeurosis-complex (TAC) and a CE compartment. TAC was inferred by the assumption that k_{MTC} consisted of an *in series* k_{CE} and TAC stiffness ($k_{TAC} = (\frac{1}{k_{MTC}} - \frac{1}{k_{CE}})^{-1}$) model assumption [1]. The dotted, black line is the linear fit of all measured k_{CE} values (Fig. 4). At 1 N, 23 N, and 30 N, we calculated Young's modulus for CE (see Eq. 2 excl. r, main text) using data from Table 1. The dotted, vertical, black lines mark 1 N and 23 N. The dashed, black line is the linear fit of all measured k_{MTC} values, and the solid, black line is the inferred k_{TAC}. 'Cross' and 'plus' are median values for passive GAS k_{MTC} and k_{CE}, respectively. 'Asterisk' is the passive muscles' estimated k_{TAC} inferred from median k_{MTC} and k_{CE}.

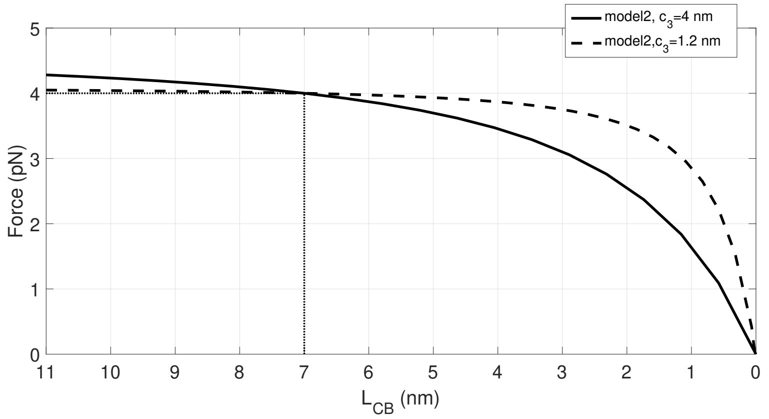

Supplementary Figure S 5 | Non-linear $F_{CB}(L_{CB})$ (*model2*). Solid line is the force for a single cross-bridge ($F_{CB,1}$) as a function of its lever arm coordinate (L_{CB}) as originally estimated [4]. Dashed line is $F_{CB,1}(L_{CB})$ as predicted for our measured impact responses (see Table 2).Horizontal, thin, dotted line and vertical, thin, dotted line indicate optimal force ($F_{CB,opt,1}$) and length ($L_{CB,opt}$), respectively, for a single cross-bridge as originally given [4].

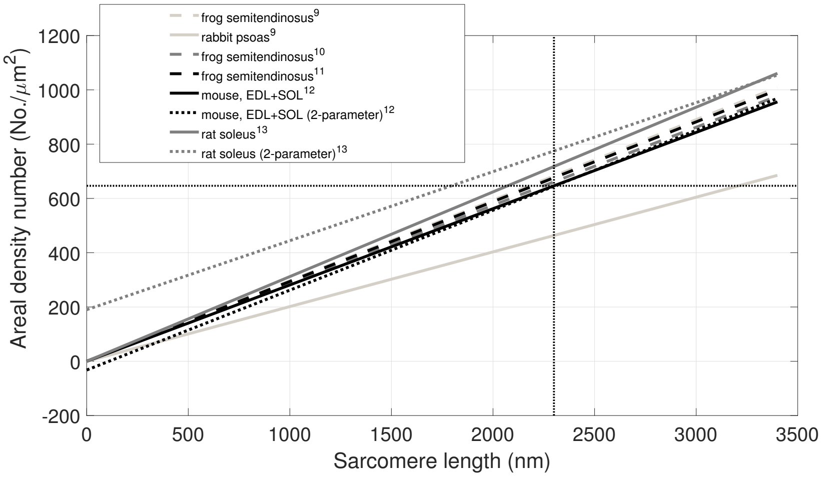

Supplementary Figure S6 | The radial areal density number of myosins in cross-sections of the A-band. The dashed, light-grey line and the solid light-grey line are 1-parameter $(a \cdot x)$ fits of frog semitendinosus and rabbit psoas data, respectively (species unknown) [18]. The dashed, grey line is the 1-parameter fit of frog, *Rana temporaria*, semitendinosus [19], and the dashed, black line is the 1-parameter fit of frog semitendinosus (species unknown) [20]. The solid, black and grey lines are 1-parameter fits to data of normal mouse soleus (SOL) combined with extensor digitorum longus (EDL) [21] and rat SOL [22], respectively (both species are unknown). The dotted lines are 2-parameter fits $(a \cdot x + b)$ to data of normal mouse EDL combined with SOL [21] and to *Sprague-Dawley* SOL [22] data, respectively. We used the radial areal density number of myosins for the combined mouse SOL and EDL data to estimate A_{hs} in this paper. We chose the 1-parameter fit of combined mouse SOL and EDL due to three criteria: I. The mouse is a mammal. II. Of all animals considered here, the mouse has the most anatomical similarities to the rat of the animals presented here. III. The 2-parameter fit to the data of the mouse is in better agreement with fibril volume constancy (intercept at 0,0) than the 2-parameter fit of the *Sprague-Dawley* SOL data. In addition, the *Sprague-Dawley* data are measured with electron micrographs instead of x-ray diffraction, which typically overestimates the radial areal density number of myosins by 10-20 % [23]. The dotted, thin, vertical line marks the optimal sarcomere length ($2.3\,\mu$m) [24,25] for a Wistar rat gastrocnemius, and the dotted, thin, horizontal line is the corresponding radial areal density number of myosins based on the 1-parameter mouse SOL+EDL fit ($1540\,\mathrm{nm}^2 = \frac{1}{649\,\mu\mathrm{m}^2}$).

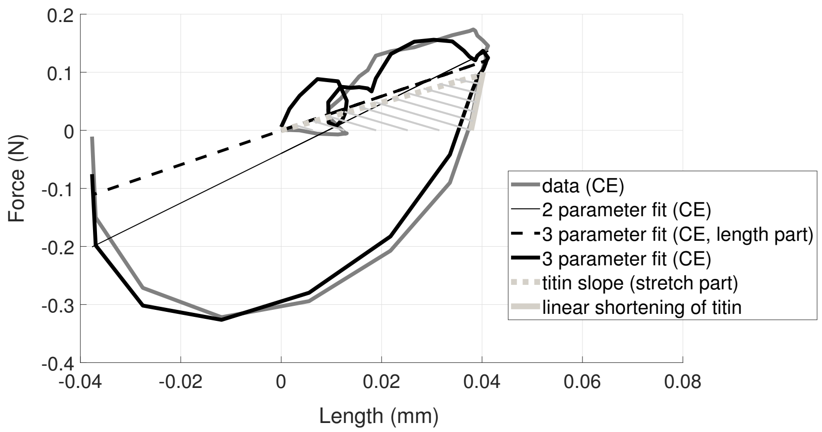

Supplementary Figure S7 | Titin contribution to CE in one passive exemplary trial. The solid, dark-grey loop is the $L_{CE,0}$ response to ΔF. The solid, thin, black line is the respective linear 2-parameter (force (length)) fit to the data. The solid, black loop depict the respective 3-parameter fit to the data, using the parameters k_i, b_i, d_i of each the function $F_i(L_i, \dot{L}_i)$ (see Eq. 1, main text), which linearly depends on length L_i and time rate of length change $\dot{L}_i$; the dashed, black line is the respective length-dependent contribution. The dotted, light-grey line shows a linear fit to isolated titin stretch data [14], and the solid, light-grey line is an idealised shortening path of the titin filament as ΔF returns to zero, for which linearity is assumed as an approximation. The work-loop is identical to Fig. 3a.

References

1. Christensen, K. B., Günther, M., Schmitt, S. & Siebert, T. Strain in shock-loaded skeletal muscle and the time scale of muscular wobbling mass dynamics. *Scientific Reports* **7**, 13266 (2017).

2. Fusi, L., Brunello, E., Reconditi, M., Piazzesi, G. & Lombardi, V. The non-linear elasticity of the muscle sarcomere and the compliance of myosin motors. *The Journal of Physiology* **592**, 1109–1118 (2014).

3. Piazzesi, G. *et al.* Skeletal muscle performance determined by modulation of number of myosin motors rather than motor force or stroke size. *Cell* **131**, 784–795 (2007).

4. Günther, M., Haeufle, D. F. B. & Schmitt, S. The basic mechanical structure of the skeletal muscle machinery: One model for linking microscopic and macroscopic scales. *Journal of Theoretical Biology* **456**, 137–167 (2018).

5. Mobley, B. A. & Eisenberg, B. R. Sizes of components in frog skeletal muscle measured by methods of stereology. *The Journal of General Physiology* **66**, 31–45 (1975).

6. Reconditi, M. Recent improvements in small angle X-ray diffraction for the study of muscle physiology. *Reports on Progress in Physics* **69**, 2709–2759 (2006).

7. Schoenberg, M. Geometrical factors influencing muscle force development. II. Radial forces. *Biophysical Journal* **30**, 69–78 (1980).

8. Lombardi, V., G, P. & Linari, M. Rapid regeneration of the actin-myosin power stroke in contracting muscle. *Nature* **355**, 638–641 (1992).

9. Powell, P. L., Roy, R. R., Kanim, P., Bello, M. A. & Edgerton, V. R. Predictability of skeletal muscle tension from architectural determinations in guinea pig hindlimbs. *Journal of Applied Physiology* **57**, 1715–1721 (1984).

10. Weis-Fogh, T. & Alexander, R. M. The sustained power output obtainable from striated muscle. In Pedley, T. J. (ed.) *Scale Effects in Animal Locomotion*, 511–526 (Academic Press, London, 1977). p. 518: $q \cdot \sigma_0$.

11. Eng, C. M. *et al.* Scaling of muscle architecture and fiber types in the rat hindlimb. *The Journal of Experimental Biology* **211**, 2336–2345 (2008).

12. Liversage, A. D., Holmes, D., Knight, P. J., Tskhovrebova, L. & Trinick, J. Titin and the sarcomere symmetry paradox. *Journal of Molecular Biology* **305**, 401–409 (2001).

13. Nishikawa, K. *et al.* Calcium-dependent titin–thin flament interactions in muscle: observations and theory. *Journal of Muscle Research and Cell Motility* published online (2019).

14. Mártonfalvi, Z. *et al.* Low-force transitions in single titin molecules reflect a memory of contractile history. *Journal of Cell Science* **127**, 858–870 (2014).

15. Rivas-Pardo, J. A. *et al.* Work done by titin protein folding assists muscle contraction. *Cell Reports* **14**, 1339–1347 (2016).

16. Tomalka, A., Weidner, S., Hahn, D., Seiberl, W. & Siebert, T. Power amplification increases with contraction velocity during stretch-shortening cycles of skinned muscle fibers. *Frontiers in Physiology* **12**, 391 (2021).

17. Lakie, M. & Campbell, K. S. Muscle thixotropy—where are we now? *Journal of Applied Physiology* **126**, 1790–1799 (2019).

18. Elliott, G. F., Lowy, J. & Worthington, C. R. An X-ray and light-diffraction study of the filament lattice of striated muscle in the living state and in rigor. *Journal of Molecular Biology* **6**, 295–305 (1963).

19. Matsubara, I. & Elliott, G. X-ray diffraction studies on skinned single fibres of frog skeletal muscle. *Journal of Molecular Biology* **72**, 657–669 (1972).

20. Brandt, P. W., Lopez, E., Reuben, J. P. & Grundfest, H. The relationship between myofilament packing density and sarcomere length in frog striated muscle. *The Journal of Cell Biology* **33**, 255–263 (1967).

21. Kurg, T., Stinson, R. H. & Millman, B. M. X-ray diffraction from striated muscles and nerves in normal and dystrophic mice. *Muscle & Nerve* **5**, 238–246 (1982).

22. Goldstein, M. A., Michael, L. H., Schroeter, J. P. & Sass, R. L. Z band dynamics as a function of sarcomere length and the contractile state of muscle. *Federation of American Societies for Experimental Biology* **1**, 133–142 (1987).

23. Millman, B. M. The filament lattice of striated muscle. *Physiological Reviews* **78**, 359–391 (1998).

24. Zuurbier, C. J., Heslinga, J. W., Lee-de Groot, M. B. E. & Van der Laarse, W. J. Mean sarcomere length-force relationship of rat muscle fibre bundles. *Journal of Biomechanics* **28**, 83–87 (1995).

25. Heslinga, J. W. & Huijing, P. A. Effects of growth on architecture and functional characteristics of adult rat gastrocnemius muscle. *Journal of Morphology* **206**, 119–132 (1990).
